# A Bayesian approach to dynamic homology of morphological characters and the ancestral phenotype of jawed vertebrates

**DOI:** 10.7554/eLife.62374

**Published:** 2020-12-04

**Authors:** Benedict King, Martin Rücklin

**Affiliations:** Naturalis Biodiversity CenterLeidenNetherlands; Chinese Academy of SciencesChina; Pennsylvania State UniversityUnited States

**Keywords:** placoderm, homology, jaws, phylogeny, vertebrate, morphology, Other

## Abstract

Phylogenetic analysis of morphological data proceeds from a fixed set of primary homology statements, the character-by-taxon matrix. However, there are cases where multiple conflicting homology statements can be justified from comparative anatomy. The upper jaw bones of placoderms have traditionally been considered homologous to the palatal vomer-dermopalatine series of osteichthyans. The discovery of ‘maxillate’ placoderms led to the alternative hypothesis that ‘core’ placoderm jaw bones are premaxillae and maxillae lacking external (facial) laminae. We introduce a BEAST2 package for simultaneous inference of homology and phylogeny, and find strong evidence for the latter hypothesis. Phenetic analysis of reconstructed ancestors suggests that maxillate placoderms are the most plesiomorphic known gnathostomes, and the shared cranial architecture of arthrodire placoderms, maxillate placoderms and osteichthyans is inherited. We suggest that the gnathostome ancestor possessed maxillae and premaxillae with facial and palatal laminae, and that these bones underwent divergent evolutionary trajectories in placoderms and osteichthyans.

## Introduction

The concept of homology underpins the cladistic analysis of morphological data. Testing of homology is usually considered a two-step process (Patterson, 1982a; Pinna, 1991). First, provisional statements of homology are made (primary homology), which are hypotheses based on comparative anatomy. Primary homologues are then subjected to cladistic analysis, and those that correspond to synapomorphies are then considered ‘secondary homologues’; this term corresponds to the vernacular use of the term homology (similarity due to common ancestry). The starting point for a cladistic analysis, the character-by-taxon matrix, is a set of primary homology statements. Primary homology statements are based upon ‘homology criteria’ (Patterson, 1988; Rutishauser and Moline, 2005). The first and most important criterion for primary homology is similarity: structures should correspond in position and structural details (developmental similarity is part of this criterion). Second is the test of conjunction: if two structures are found together on a single animal, they cannot be homologous (Patterson, 1988).

**Figure 1.**
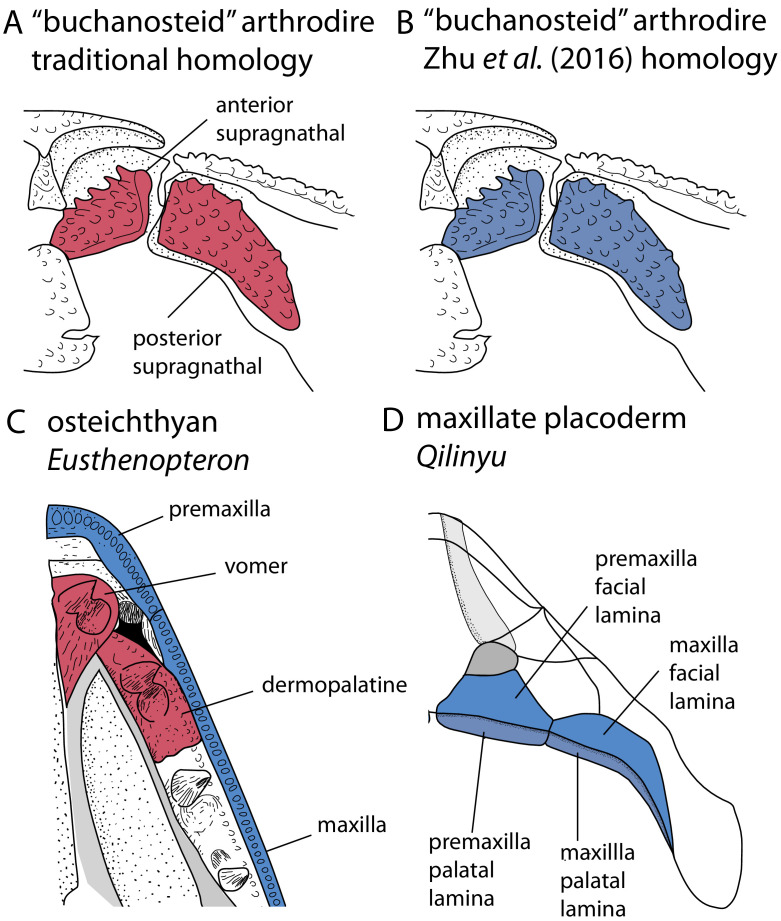
Upper jaw bones in arthrodire placoderms, maxillate placoderms and osteichthyans, showing alternative homology assignments for the arthrodiran supragnathals. (**A–B**) Arthrodire in palatal view, showing anterior and posterior supragnathals. Based on Hu et al., 2017. (**C**) Osteichthyan *Eusthenopteron* in palatal view, based on Jarvik, 1980. (**D**) Maxillate placoderm *Qilinyu* in palatal view, based on Zhu et al., 2016. Blue coloration indicates the premaxilla-maxilla series, red coloration indicates the vomer-dermopalatine series. The alternative coloration of arthrodire supragnathals in A and B represents the alternative homology statements for these bones (homology states 0 and 1 respectively).

Placoderms are stem gnathostomes, and the evolution and morphology of their jaws is thus of particular interest. The upper jaw bones of placoderms present a major unresolved example of a homology problem. Arthrodiran placoderms possess two upper gnathal plates in their jaws, termed the anterior and posterior supragnathals (Figure 1A). These have traditionally been considered primary homologues of the vomers and dermopalatines of osteichthyans (Stensiö, 1963a; Stensiö, 1969), which are palatal bones sitting on the roof of the mouth, inside the maxilla and premaxilla (Figure 1C). This proposed homology of placoderm supragnathals and osteichthyan palatal bones is based on positional criteria.

The discovery of maxillate placoderms reignited debates about the homology of placoderm and osteichthyan skull bones (Zhu et al., 2013; Zhu et al., 2016), and a new hypothesis regarding the homology of arthrodiran gnathal plates was proposed (Zhu et al., 2016; Zhu et al., 2019). Maxillate placoderms have premaxillae and maxillae with both palatal and facial laminae (Figure 1D). The palatal laminae articulate with the ventral surface of the braincase, and therefore correspond in position to arthrodiran supragnathals. The facial laminae are continuous with the external dermal bones of the skull, and are equivalent in position to osteichthyan premaxillae/maxillae. Zhu et al., 2016 therefore proposed the homology of arthrodiran supragnathals with the premaxilla and maxilla of osteichthyans. This negates a putative homology with the osteichthyan vomer-dermopalatine series, which would otherwise fail the test of conjunction (placoderm supragnathals cannot be homologous to both the premaxilla-maxilla and vomer-dermoplatine series). Nevertheless, the traditional hypothesis for the homology of arthrodiran supragnathals continues to be discussed in the literature (Hu et al., 2017). There are therefore two opposing possibilities for the primary homology of arthrodiran gnathal bones.

A number of approaches have been proposed to distinguish between conflicting hypotheses of primary homology. Jardine, 1969 provided a method that selected between alternative homologies of rhipidistian skull roof bones without reference to phylogeny, based on the criterion of preservation of spatial relationship. Lee, 1998 used parsimony to distinguish between conflicting conjectures of homology on a fixed tree topology. The latter was essentially the approach taken by Zhu et al., 2016 to support their hypothesis regarding placoderm supragnathal bones. However, choices regarding primary homology statements necessarily restrict the search for secondary homologues: phylogenetic analyses can only find the optimal tree given the input character matrix. Indeed, it has been suggested that the two-step approach to homology entails a degree of circularity (Rieppel, 1996), although this is likely to only be an issue when a phylogeny is weakly supported. A solution to this issue is the simultaneous inference of primary and secondary homology, termed *dynamic homology.*

Dynamic homology of molecular sequence data in a parsimony framework has been implemented in the software POY (Wheeler et al., 2006; Varón et al., 2010). Models for dynamic homology of molecular data have also been developed (Lunter et al., 2005; Redelings and Suchard, 2005; Wheeler, 2006) and implemented within the phylogenetic software Bali-Phy (Suchard and Redelings, 2006) and POY 5.0 (Wheeler et al., 2015). Agolin and D’Haese, 2009, used the parsimony implementation in POY to analyze morphological data (specifically the setae of collembolans). However, morphological characters, with their hierarchical dependence relationships and arbitrary sequence within a data matrix, are often not amenable to models used to align molecular data. Ramírez, 2007 presented a parsimony approach to dynamic homology, using the empirical example of sclerites on the male copulatory organs of anyphaenid spiders. In this method, multiple matrices with alternative alignments of morphological characters were analysed, and the phylogenetic tree and homology combination with the shortest tree length was selected.

Dynamic homology methods for morphological data have thus far been rarely explored, and are restricted to parsimony-based approaches. However, a Bayesian approach would confer a number of advantages. Alternative homology statements could be considered as ‘nuisance parameters’, such that phylogenetic trees could be estimated while accounting for uncertainty in primary homology statements. Conversely, if discovering homology is the aim, the tree topology could be considered the ‘nuisance parameter’. Bayesian tip-dated analysis of morphological data allows comparative analysis (such as biogeography or ancestral state reconstruction) to occur simultaneously with tree search (e.g. Lee et al., 2018). Comparative analyses could therefore be performed while accounting for uncertainty in both tree topology and primary homology statements.

Here, we present an approach to dynamic homology within a Bayesian tip-dating framework, which we use to test the alternative conjectures of placoderm jaw bone homologies. The homology relations of placoderm jaw bones have implications for our understanding of character evolution in early vertebrates. In particular, homology of placoderm supragnathal bones with the marginal jaw bones of osteichthyans suggests a deep (early) origin for these bones. Zhu et al., 2016 proposed their hypothesis within the framework of placoderm paraphyly (Brazeau, 2009; Davis et al., 2012; Zhu et al., 2013), but an alternative hypothesis of placoderm monophyly (excluding maxillate placoderms) is supported by an essentially equivalent amount of morphological data, and is strongly supported under Bayesian tip-dated methods (King et al., 2017). The implications of the hypothesis of Zhu et al., 2016 within the framework of placoderm monophyly have not been discussed. We therefore simultaneously estimated a credible set of phenotypes for the (apomorphy-defined) gnathostome common ancestor to explore character evolution in early gnathostomes while accounting for phylogenetic uncertainty, divergence date uncertainty, and alternative placoderm jaw bone homologies.

### Dynamic homology

**Figure 2.**
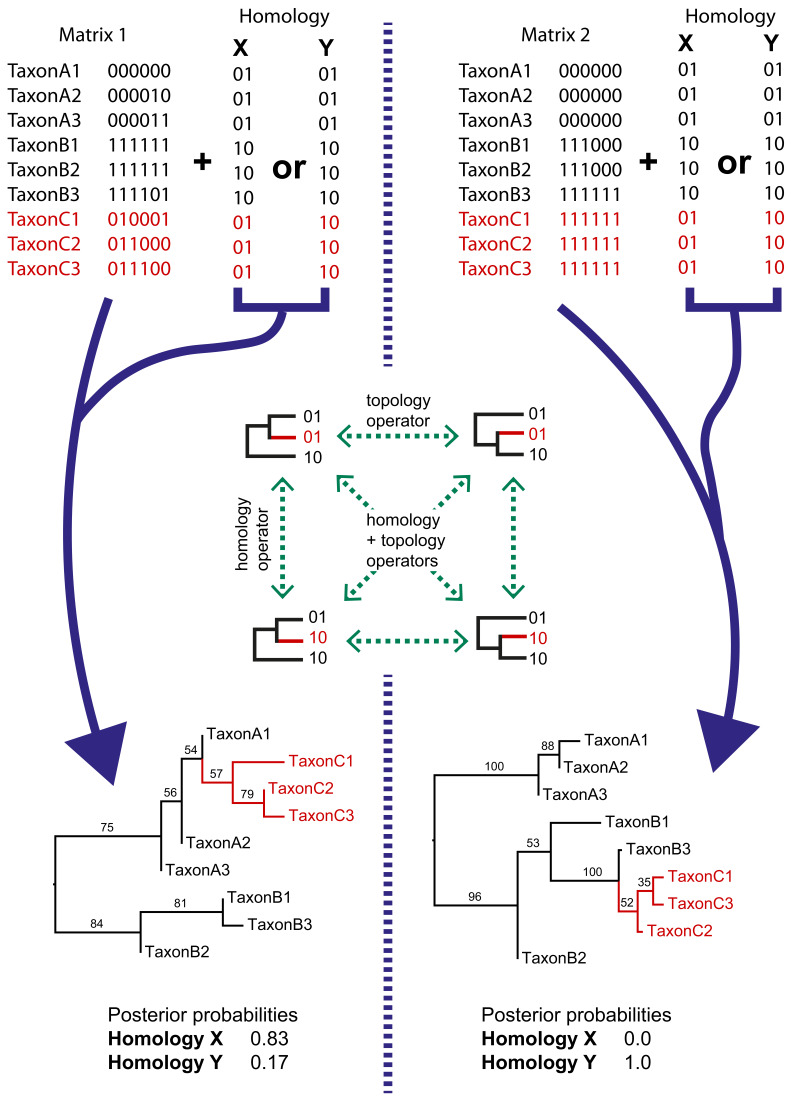
Simple examples of dynamic homology applied to matrices with six characters with fixed homology and two with estimated homology. Taxa C1–3 have alternative homologies (homology X and Y). For matrix1, there is moderate support for group C to fall within group A, leading to a higher posterior probability for homology X than homology Y. In matrix 2, there is strong support for taxon group C to fall within group B, leading in turn to strong support for homology Y.

We implemented a method for dynamic homology of morphological characters within the open source BEAST2 software package *homology* (https://github.com/king-ben/homology; King, 2021; copy archived at swh:1:rev:6e6dbd77443b0d963640b3cb603c4310b5a4b47e). The method takes as inputs alternative character coding alignments, here called *homology alignments*, which are alternative character codings corresponding to alternative homology hypotheses for morphological features (for example placoderm jaw bones). Homology alignments can be included alongside fixed alignments (Figure 2), such that only a subset of characters has dynamic homology. During a BEAST2 MCMC run, the homology alignment used to calculate the posterior is determined by a homology state parameter, which is changed by an operator (Figure 2). The MCMC will spend more time in the homology state corresponding to the homology alignment that returns the highest tree likelihood.

**Figure 3.**
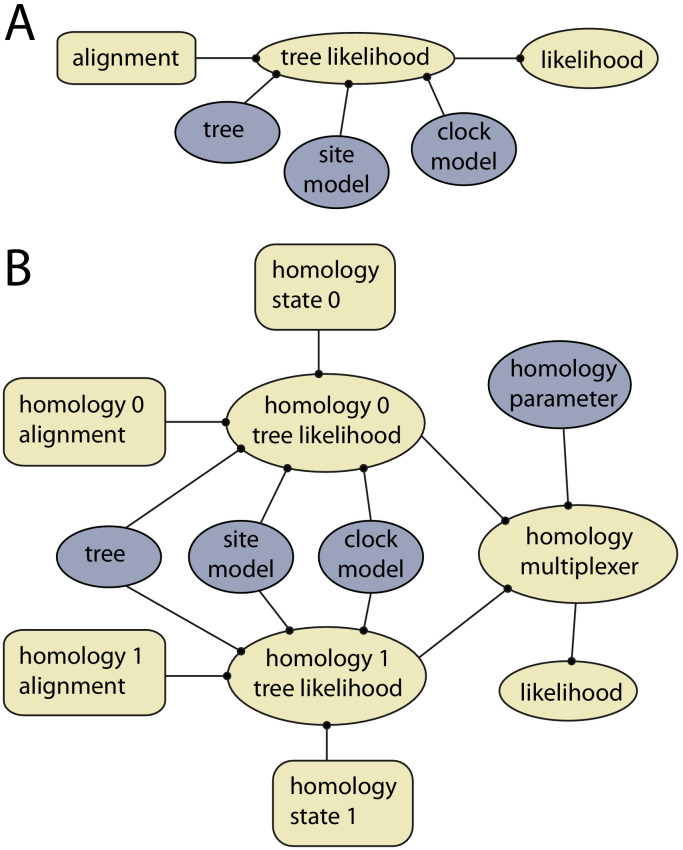
Example of models with and without dynamic homology of morphological characters. (**A**) Model diagram of a fixed homology partition. Tree likelihood takes as input a data alignment, tree, site model and clock model, and the calculated tree likelihood is passed to the likelihood, where it is combined with other partitions. Rectangles indicate fixed inputs, whereas ovals are model components that change during the MCMC. Blue shaded components are changed by operators, either directly (tree) or indirectly (site model, clock model). (**B**) Model diagram for a partition with dynamic homology with two homology states. The homology-multiplexer passes either the value of homology tree likelihood 0 or homology tree likelihood 1 to the likelihood, depending on the current value of the homology parameter.

The *homology* package contains two java classes corresponding to CalculationNodes (which calculate a part of the posterior based on inputs). These are *HomologyTreeLikelihood* and *HomologyMultiplexer* (Figure 3). The *HomologyTreeLikelihood* class is an extension of the core BEAST2 *TreeLikelihood* class, and differs in associating a particular homology alignment with a homology state. The *HomologyMultiplexer* takes as input two or more HomologyTreeLikelihoods and a *homology parameter*, the latter is an integer parameter with states (0, 1,…,N) corresponding to N homology states (one for each HomologyTreeLikelihood). During an MCMC run, the homology-multiplexer returns the value of the homology tree likelihood corresponding to the current state of the homology parameter. Due to the possibility of correlated tree- and homology-space, the package also contains two updated tree operators which simultaneously change the tree topology and homology state: *HomologySAWilsonBalding* and *HomologySAExchange*.

## Results

**Figure 4.**
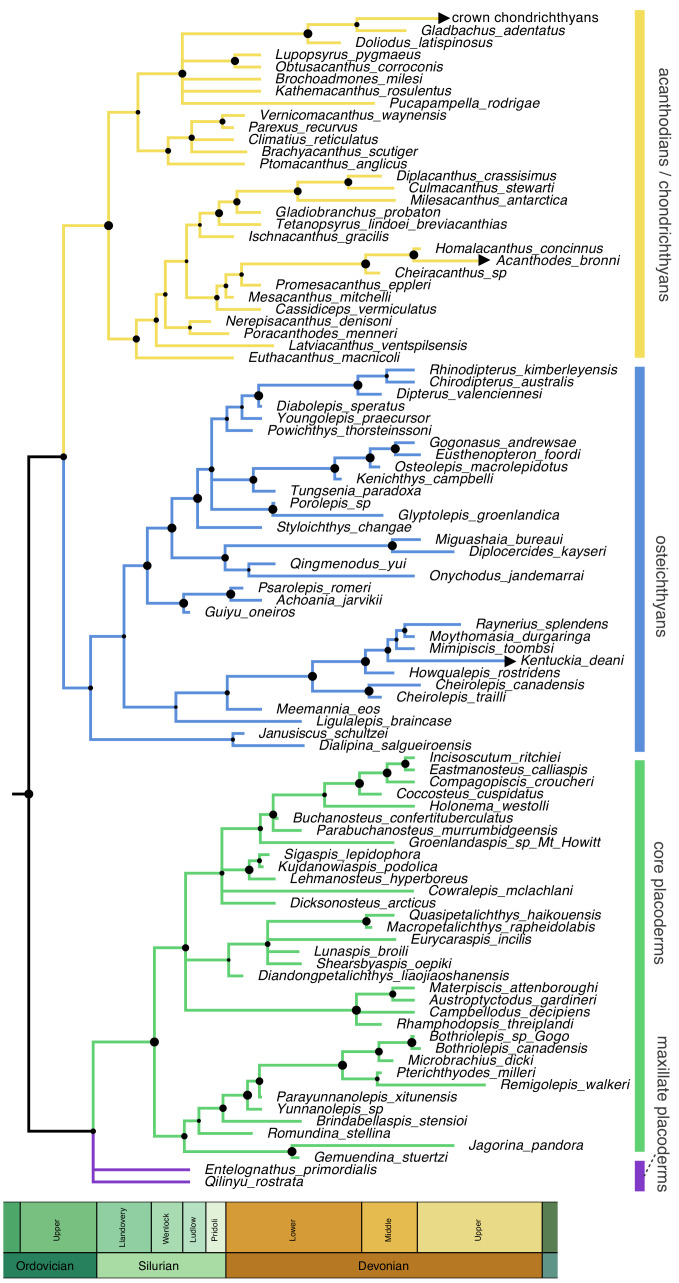
Time-scaled 50% majority-rule consensus tree from tip-dated homoplasy-partitioned analysis of gnathostome fossils, with dynamic homology of upper jaw bones in placoderms. Node circles indicate posterior probabilities. Branches with arrowheads (crown chondrichthyans, *Acanthodes*, *Kentuckia*) indicate tip age(s) are younger than the range displayed in the figure.

Homoplasy-partitioned Bayesian tip-dated analysis (with dynamic homology of placoderm upper jaw bones) of the gnathostome fossil dataset results in the majority-rule consensus tree shown in Figure 4. Core placoderms (placoderms excluding maxillate forms) are monophyletic (posterior probability, pp = 1.0). The maxillate placoderms *Entelognathus* and *Qilinyu* are resolved as the sister group to core placoderms, but with weak support (pp = 0.70). *Janusiscus* is resolved as a stem osteichthyan, sister to *Dialipina*, but support for this grouping is again weak (pp = 0.57).

**Figure 5.**
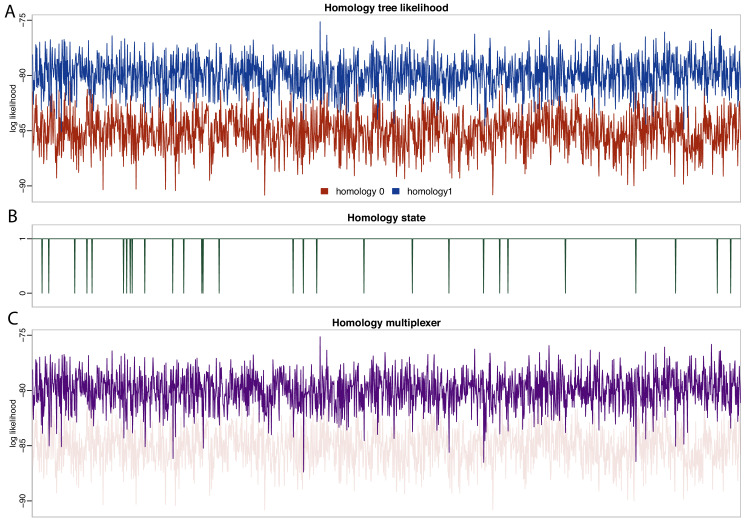
Likelihood and parameter traces during BEAST2 MCMC, with dynamic homology of placoderm jaw bones. (**A**) Tree likelihoods using homology alignment 0 (placoderm supragnathals are vomers/dermopalatines) are lower than those for homology alignment 1 (placoderm supragnathals are premaxillae/maxillae). (**B**) The MCMC only rarely samples homology state 0. (**C**) The homology-multiplexer therefore largely returns the tree likelihood of homology alignment 1 (homology tree likelihood 0 is replotted with transparency for reference).

We find strong support for homology state 1 (pp = 0.984), corresponding to the hypothesis that placoderm supragnathal bones are homologous to premaxillae and maxillae (Zhu et al., 2016). The mean log likelihood for homology alignment 0 is −85.099, and for homology alignment 1 –79.883. The MCMC chain therefore rarely accepts proposals for homology state 0 (Figure 5).

**Figure 6.**
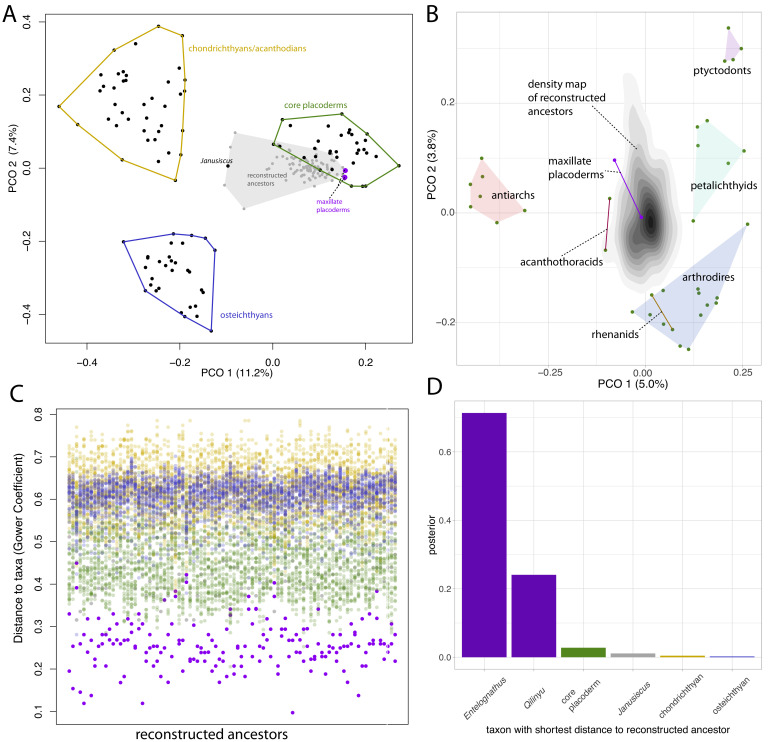
Distance plots suggest placoderms, and particularly maxillate placoderms are the gnathostomes least divergent from the gnathostome ancestor. (**A**) PCO plot of all gnathostome taxa in the matrix (black points) and a sample of 90 reconstructed ancestors (gray points and shaded convex hull). (**B**) PCO plot of placoderm taxa and reconstructed ancestors, with the latter point cloud converted to a density plot. (**C**) Each column represents a reconstructed ancestor (n = 90), with gnathostome fossils plotted with y-axis coordinates corresponding to distance from each reconstructed ancestor. (**D**) Frequency plot of the taxon with the shortest distance to the reconstructed ancestor across the whole posterior sample (n = 1801). Core placoderm, osteichthyan and chondrichthyan (including acanthodian) taxa are combined into single bins.

Principal coordinates (PCO) analysis of gnathostome fossils reveals chondrichthyans (including acanthodians), osteichthyans and core placoderms form three discrete and well-separated groups (Figure 6A), concordant with the results of Davis et al., 2012. *Janusiscus* is an outlier, lying equidistant from the three groups, whereas maxillate placoderms plot close to core placoderms.

We used ancestral sequence logging in BEAST2 to reconstruct the phenotype of the gnathostome ancestor in each sample from the posterior. A sample of 90 of these reconstructed ancestors included in the PCO mostly plot close to placoderms, with a small number plotting in outlier positions closer to *Janusiscus*. A second PCO using only placoderms (maxillate and core) and the reconstructed ancestors is shown in Figure 6B, with the point cloud of reconstructed ancestors converted to a 2D density plot. *Entelognathus* plots close to the center of the ancestral area, while *Qilinyu*, arthrodires, petalichthyids and acanthothoracids are equidistant. Antiarchs and ptyctodontids plot the furthest from the reconstructed ancestors. However, it should be noted that the two principal axes account for less than 10% of the total variance.

Plotting the raw distance measures shows that maxillate placoderms are the most similar taxa to the reconstructed ancestors (Figure 6C). The individual taxon with the lowest distance to the reconstructed ancestor (in each sample from the posterior, n = 1801) was a maxillate placoderm for 95% of the reconstructed ancestors (Figure 6D). This suggests that of the known gnathostome fossils, the maxillate placoderms (in particular *Entelognathus*) are the least divergent known descendants of the gnathostome common ancestor.

The reconstructed ancestors also allow us to calculate the posterior probability of particular character states at the gnathostome node (i.e. the proportion of reconstructed ancestors with a particular character state). Table 1 displays a number of characters of interest, including characters of the upper jaw bones and characters possessed by some core placoderms, argued to be retained plesiomorphies under the hypothesis of placoderm paraphyly (Brazeau, 2009; Dupret et al., 2014). Results for all characters are available in the supplementary information (Table 1; Source data 1). Our results suggest that the gnathostome ancestor had a premaxilla and maxilla with both palatal and facial laminae, no vomer-dermopalatine series, anterior/ventral nasal capsules and lateral orbits not surrounded by neurocranium. Putative core placoderm synapomorphies (claspers, optic fissure) are reconstructed as absent at the gnathostome node with moderate support (Table 1). This uncertainty is likely due to the high proportion of missing data for these characters. Critically, it is unknown whether or not maxillate placoderms possessed these putative core placoderm synapomorphies.

**Table 1.** Character states reconstructed at the common ancestor of apomorphy-defined gnathostomes.

Character	Reconstructed ancestral state	Posterior probability
Premaxilla	Present	1.0
Maxilla	Present	0.96
Facial laminae	Present	0.96
Palatal laminae	Present	0.93
Vomer	Absent	0.93
Dermopalatine	Absent	0.95
Nasal capsules	Anterior/ventral	0.94
Orbit dorsal, surrounded by neurocranium	Absent	0.96
Claspers	Absent	0.79
Optic fissure	Absent	0.78

## Discussion

**Figure 7.**
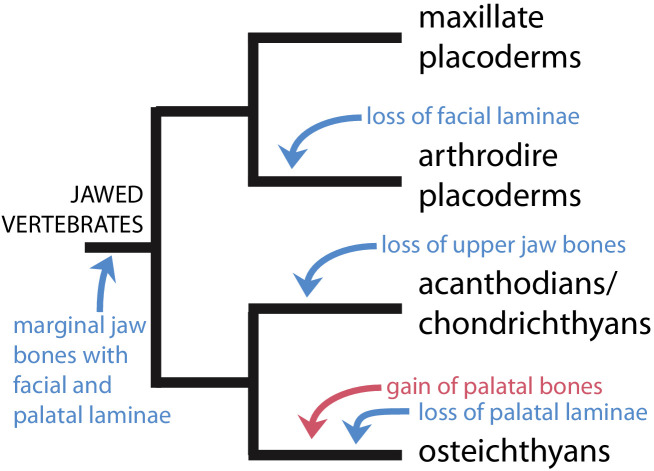
Scenario for the evolution of upper jaw bones in gnathostomes (jawed vertebrates). Red arrow indicates change to the palatal (vomer-dermopalatine) series of dermal jaw bones, blue arrows indicate changes to the marginal (premaxilla-maxilla) series.

We find strong support for the hypothesis of Zhu et al., 2016, that placoderm supragnathal bones are homologous to the maxilla and premaxilla of osteichthyans and maxillate placoderms (Figure 5). However, we present a distinct scenario regarding the trajectory of upper jaw bone evolution (Figure 7). Zhu et al., 2016 proposed that the plesiomorphic states of the maxillae and premaxillae were as palatal bones, exemplified by the arthrodiran condition. Facial laminae were then gained in the common ancestor of maxillate placoderms and crown gnathostomes, and palatal laminae were lost in osteichthyans. We instead propose that the common ancestor of (apomorphy-defined) gnathostomes possessed maxillae and premaxillae with both facial and palatal laminae. Facial laminae were subsequently lost in core placoderms and palatal laminae were lost in osteichthyans. The stem osteichthyans *Lophosteus* and *Andreolepis* show a possibly intermediate condition, in which the marginal jaw bones have internal (oral or palatal) laminae that are more strongly developed compared to other osteichthyans (Botella et al., 2007; Cunningham et al., 2012; Chen et al., 2016; Chen et al., 2020).

In concordance with Zhu et al., 2016, we find strong support for a lack of the vomer-dermopalatine series in the gnathostome ancestor. Our scenario suggests that arthrodires, for which morphological data of the jaws is best known (Hu et al., 2017), exhibit a specialized condition. Independent evidence for this hypothesis comes from recently described acanthothoracids (Vaškaninová et al., 2020), which exhibit marginal dentitions and jaw bones quite unlike those of arthrodires. In addition, the inner dental arcade of the stem osteichthyan *Lophosteus* consists of many ‘tooth cushions’ bearing no resemblance to arthrodire gnathal plates (Chen et al., 2017).

The divergent trajectories of the premaxilla and maxilla in osteichthyans and core placoderms may be associated with alternative ecological roles among their earliest members. Osteichthyans from the Silurian Kuanti formation include the large *Megamastax* (Choo et al., 2015). The maxillate placoderms from the same formation however are clearly not apex predators, lacking large teeth on their jaw bones and in the case of *Entelognathus*, possess immovable eyes (Zhu et al., 2013). The loss of facial laminae in core placoderms may be associated with increased focus on crushing invertebrate prey, and may be analogous to the loss of the maxilla and specialization of the vomers in lungfishes. Conversely, the predatory osteichthyans emphasized the external tooth row and thus facial laminae.

Homology of the arthrodiran supragnathals with the premaxillae and maxillae of maxillate placoderms is consistent with observations from comparative anatomy (Zhu et al., 2016; Zhu et al., 2019). The snouts of maxillate placoderms differ from those of arthrodires mainly in the degree of dermal bone cover and are very similar in terms of their gross morphology. An early arthrodiran snout, such as that of *Kujdanowiaspis* (Dupret, 2010) differs from the maxillate placoderm condition by absence of facial laminae and a relatively small internasal plate compared to the large anterior premedian plate of *Entelognathus* (Zhu et al., 2013). Zhu et al., 2019 suggested that the arthrodiran condition results from the inward shift of the upper jaw bones. However, the downturned, ventrally directed, snouts of maxillate placoderms means that reduction of the facial laminae and premedian plate are the only transformations required to leave the upper jaw bones separated from the dermal skull roof and in a palatal position, as in arthrodires.

The results of our phenetic analysis of reconstructed ancestors suggest maxillate-placoderm-like conditions in the last common ancestor of (apomorphy-defined) gnathostomes. Due to the nested position of acanthothoracids and antiarchs within a monophyletic core placoderms, we find strong support for anterior-ventral nasal capsules and lateral eyes in the gnathostome ancestor (Table 1). Under this hypothesis, the dorsal nasal capsules of antiarch, acanthothoracid and rhenanid placoderms are convergent with those of the jawless osteostracans and galeaspids, rather than representing shared plesiomophies (King et al., 2017). Conversely, the shared cranial architecture of arthrodires, maxillate placoderms and osteichthyans (Dupret et al., 2014), represent shared plesiomorphies (Table 1; King et al., 2017). Within agnathan fishes, the braincase proportions of the jawless heterostracans, which probably possess paired anterior nasal capsules (Halstead, 1973; Janvier, 1996), may represent the plesiomorphic gnathostome condition more closely than osteostracans or galeaspids.

Although our phenetic analysis suggests that maxillate placoderms are the gnathostomes morphologically closest to the ancestral condition, we are not suggesting that they are directly ancestral. The distance from each reconstructed ancestor is usually in the range 0.2–0.3, suggesting that even maxillate placoderms are highly derived from the gnathostome common ancestor. This result is not surprising given that our analysis suggests gnathostomes diverged during the Ordovician (Figure 4). Tentative support for this divergence might be found in the enigmatic fossils of *Skiichthys* (Smith and Sansom, 1997) and Mongolepidae (suggested to be early chondrichthyans, Andreev et al., 2016). Maxillate placoderms are never recovered as sampled ancestors in the analysis, and the fact that they are of the same age as the osteichthyan *Guiyu* (Zhu et al., 2009) precludes this. *Entelognathus* and *Qilinyu* are themselves quite disparate and possess their own specializations, most notably the eyes of *Entelognathus* (Zhu et al., 2013; Zhu et al., 2016).

The results of our analysis are contingent on a phylogenetic hypothesis, in particular the monophyly of core placoderms, which is only strongly supported under a Bayesian tip-dating approach. The differences between parsimony and Bayesian tip-dated trees are discussed at length in King et al., 2017. The hypothesis of placoderm paraphyly (Brazeau, 2009; Davis et al., 2012; Zhu et al., 2013), implies a radically different scenario for character evolution (Dupret et al., 2014), in which the maxillate placoderms are not representative of ancestral conditions.

Our study proposes the application of dynamic homology concepts to morphological characters in a Bayesian framework. In this manuscript we have applied the method to placoderm jaw bones, but it could also potentially be used to examine skull roof homologies in the future. It should be noted that the simultaneous analysis of primary and secondary homology has been criticized (Simmons, 2004), because adding new morphological characters to a data matrix should be a test of phylogenetic relationships, rather than simply adding further support to a given phylogenetic hypothesis. Thus, it can be argued that multiple conflicting primary homology statements should only be analysed with dynamic homology when they are equally plausible. In such cases, supporting the primary homology statement that best fits a phylogenetic hypothesis is preferable to an arbitrary choice. There may also exist cases where alternative primary homology statements support different tree topologies, and in this case arbitrary choices of primary homology statements could lead to suboptimal phylogenetic trees.

## Materials and methods

We compiled a morphological data matrix of gnathostome fossils (Supplementary file 1). The matrix is based on King et al., 2017 with a revised taxon and character matrix. The taxon list was updated with the addition of *Gladbachus adentatus*, *Milesacanthus antarctica*, *Nerepisacanthus denisoni*, *Rhinodipterus kimberleyensis*, *Chirodipterus australis*, *Dipterus valenciennesi*, *Tungsenia paradoxa*, *Diplocercides kayseri*, *Qingmenodus yui*, *Raynerius splendens*, *Lehmanosteus hyperboreus*, *Shearsbyaspis oepiki*, and *Qilinyu rostrata. Ramirosuarezia boliviana*, *Wuttagoonaspis fletcheri*, *Gavinaspis convergens* and *Osorioichthys marginis* were removed.

Characters concerning the premaxillae, maxillae, dermopalatines and vomers were coded into two alternative *homology alignments*. These characters included presence and absence of these bones, as well as dependent characters. One alignment (homology state 0) was coded according the traditional interpretation of placoderm jaw bones (Figure 1A), in which the placoderm supragnathal bones are considered primary homologues of the vomer-dermopalatine series of osteichthyans. A second alignment (homology state 1) was coded according to the alternative interpretation (Zhu et al., 2016), in which placoderm supragnathal bones are considered primary homologues of the premaxilla-maxilla series of osteichthyans and maxillate placoderms. In total, the matrix had 489 characters with fixed homology, and 18 with variable homology.

We analysed the matrix in BEAST2.6.2 (Bouckaert et al., 2019), using the beagle calculation library (Ayres et al., 2019). We used homoplasy-based partitioning (Rosa et al., 2019) to account for rate variation among characters. Homoplasy was calculated using an implied weights parsimony analysis in TNT (Goloboff and Catalano, 2016), with concavity constant k = 10. Characters with different homoplasy values depending on homology state were assigned the lower value. Characters were partitioned according to the number of states as well as homoplasy. Each partition was assigned a separate mutation rate parameter and was analysed using the Mk substitution model (Lewis, 2001). The weighted mean value of the mutation rates was fixed at one, and each individual mutation rate parameter was assigned a normal distribution prior, with mean one and standard deviation 2.

We implemented a sampled ancestor birth-death model (Gavryushkina et al., 2014). The birth rate was assigned a lognormal prior with mean (in real space) 0.14 and standard deviation 0.9. Extinction and sampling rates were assigned exponential priors with mean 0.1. Tip dates were assigned to fossil sites with uniform priors on fossil site ages (King and Rücklin, 2020). Gnathostomes, gnathostomes+osteostracans and polybranchiaspids were constrained to be monophyletic. The clock model was an uncorrelated lognormal relaxed clock (Drummond et al., 2006) with a lognormal prior (mean −5.5, standard deviation 2) on clock rate and an exponential prior (mean 1) on clock standard deviation. We used ancestral sequence logging to reconstruct ancestral states for all characters at the (apomorphy-defined) gnathostome node at every sampled generation of the MCMC. This leads to 1801 ‘reconstructed ancestors’, which comprise a credible set of phenotypes at the gnathostome crown node.

We ran the analysis for 800 million generations, and for four independent runs. The MCMC chain was sampled every 400000 generations, and 10% of the run was discarded as burn-in, resulting in a posterior sample of 1801 trees. Convergence of 4 independent runs was confirmed in Tracer 1.7 (Rambaut et al., 2018) and RWTY (Warren et al., 2017). Following the recommendations of O'Reilly and Donoghue, 2018, we calculated the 50% majority-rule tree in the R package ape (Paradis and Schliep, 2019), then time-scaled and annotated this tree using TreeAnnotator 1.10.2 (Suchard et al., 2018). The Beast2 xml file is available in the supplementary information (Supplementary file 2).

We used distance-based methods to determine the similarity of known fossil taxa to the reconstructed sequences at the gnathostome node. Principal coordinates analysis was performed in the package Claddis (Lloyd, 2016) in R 4.0.0 ‘Arbor Day’ (R Development Core Team, 2018). We used the Maximum-Observable Rescaled Distance, equivalent to the Gower, 1971 coefficient for our dataset. First, we performed ordination using the gnathostome fossils in our dataset, and a sample of the reconstructed ancestors from BEAST2 (Figure 6A). This sample consisted of 5% of the posterior sample, from which we excluded those sampled generations where the homology state was 0 (n = 1), for a total of 90 reconstructed ancestors. Homology alignment 1 was used for distance calculations. A second ordination was performed using only placoderms (both core placoderms and maxillate placoderms)(Figure 6B). The point cloud of reconstructed ancestors was converted to a density plot using ggplot (Wickham, 2016). We also plotted the raw distance measures of each gnathostome taxon to each of the 90 reconstructed ancestors (Figure 6C). Finally, we calculated the taxon with the shortest distance to the reconstructed ancestor for the entire posterior distribution (1801 reconstructed ancestors). These calculations used the homology alignment corresponding to the sampled homology state.

## Figures and Tables

**Appendix 1—figure 1.**
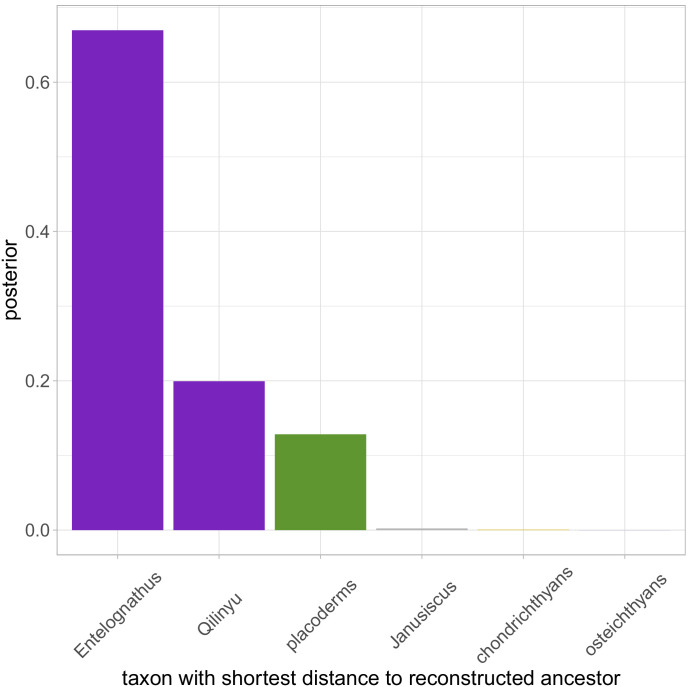
Frequency plot of the taxon with the shortest distance to the reconstructed ancestor across the whole posterior sample (n = 1801), when data is analysed with a Silurian age for *Yunnanolepis*.

## Data Availability

The data matrix in nexus format and the BEAST2 xml file are available in the supplementary information. The beast2 source code and R analysis scripts are available at https://github.com/king-ben/homology (copy archived at https://archive.softwareheritage.org/swh:1:rev:6e6dbd77443b0d963640b3cb603c4310b5a4b47e). The following datasets were generated:

## Additional files

Source data 1. Character state probabilities at the (apomorphy-defined) gnathostome node for all characters.

Supplementary file 1. Data matrix in nexus format.

Supplementary file 2. Beast2 xml file.

Transparent reporting form

## Appendix 1

Sensitivity analysis Bayesian tip-dated analysis may be sensitive to incomplete taxon sampling (O'Reilly and Donoghue, 2020). The fossil record of early gnathostomes may be biased by the nearshore origination of the major groups (Sallan et al., 2018). One example of a possible bias are the antiarchs. The earliest antiarch included in our dataset is the Lochkovian *Yunnanolepis*. However, the antiarch *Shimenolepis* is known from the Silurian (Ludlow) of China, although its fragmentary remains provide few characters for phylogenetic analysis. To test the effect of Silurian antiarchs on our results we reanalyzed the data with a Ludlow age assigned to *Yunnanolepis*. The major results of the analysis were unchanged, although there was a slight increase in uncertainty. Core placoderm monophyly was supported (pp = 0.98, down from 1.0), with maxillate placoderms as sister group to core placoderms (pp = 0.52, down from 0.70). Homology of arthrodire gnathal plates and the premaxilla/maxilla was supported (pp = 0.98, down from 0.984). Phenetic analysis supported maxillate placoderms as the least diverged known gnathostomes (pp = 0.87, down from 0.95). There was increased support for a member of the core placoderms being the least diverged gnathostome (Appendix 1—figure 1), with *Diandongpetalichthys* accounting for most of that probability. Support for key character states at the gnathostome node was slightly reduced (Appendix 1—table 1). Overall, this sensitivity shows that our conclusions are robust to at least some issues regarding fossil sampling. However, future studies should aim to further explore the effect of taxon sampling on results.

**Appendix 1—table 1.** Probabilities of key character states at the gnathostome node, when data is analysed with a Silurian age for *Yunnanolepis*.

Character	Reconstructed ancestral state	Posterior probability
Premaxilla	Present	0.97
Maxilla	Present	0.92
Facial laminae	Present	0.89
Palatal laminae	Present	0.97
Vomer	Absent	0.96
Dermopalatine	Absent	0.88
Nasal capsules	Anterior/ventral	0.85
Orbit dorsal, surrounded by neurocranium	Absent	0.96
Claspers	Absent	0.76
Optic fissure	Absent	0.69

## Appendix 2

Sources for taxa and age ranges Hemicyclaspis murchisoni Stensiö, 1932 Shropshire Downtownian. Pridoli, 423–419.2 Ma. Cephalaspis lyelli Stensiö, 1932; White, 1958 Lower Old Red Sandstone, Glammis. Lochkovian, 419.2–410.8 Ma. Zenaspis salweyi Stensiö, 1932 Lower Old Red Sandstone. Skirrid Fawr, Senni/St Maughans Formation. Lochkovian, 419.2–410.8 Ma. Benneviaspis holtedahli Janvier, 1985a Ben Nevis Formation, Red bBay Group. Late Lochkovian, 413.6–410.8 Ma. Boreaspis macrorhynchus Janvier, 1985a Wood Bay Formation. Early Pragian, 410.8–409.7 Ma. Norselaspis glacialis Janvier, 1981 Wood Bay Formation. Early Pragian, 410.8–409.7 Ma. Nectaspis areolate Wängsjö, 1952, (Janvier, 1981) Wood Bay Formation. Late Pragian, 408.7–407.6 Ma. Procephalaspis oeselensis Robertson, 1939; Denison, 1951; Janvier, 1985b Saaremaa. Ludlow, 427.4–423 Ma. Tremataspis mammillata Robertson, 1938a; Robertson, 1938b; Denison, 1947; Denison, 1951; Janvier, 1985b Saaremaa. Ludlow, 427.4–423 Ma. Waengsjoeaspis excellens Wängsjö, 1952; Janvier, 1985a Fraenkelryggen Formation. Late Lochkovian, 413.6–410.8 Ma. Escuminaspis laticeps Janvier et al., 2004 Escuminac Formation. Early Frasnian, 382.7–379.2 Ma. Eugaleaspis changi Liu, 1965; Zhu and Gai, 2007 Xitun Formation, Liaojaoshan. Late Lochkovian, 413.6–410.8 Ma. Hanyangaspis guodingshanensis Zhu and Gai, 2007 Guodingshan Formation. Telychian, 438.5–433.4 Ma. Polybranchiaspis liaojiaoshanensis Liu, 1965; Liu, 1975 Xishancun and Xitun Formations. Lochkovian, 419.2–410.8 Ma. Bannhuanaspis vukhuci Janvier et al., 1993 Bac Bun Formation. Late Lochkovian–early Pragian, 413.6–409.7 Ma. Wenshanaspis zhichangensis Zhao et al., 2001 Posongchong Formation, Wenshan. Pragian, 410.8–407.6 Ma. Shuyu zhejiangensis Gai et al., 2011 Maoshan Formation. Late Telychian–early Wenlock, 435.1–431.4 Ma. Polybranchiaspid sp histological samples Wang et al., 2005 Xishancun and Xitun Formations. Lochkovian, 419.2–410.8 Ma. *Yunnanolepis* sp Zhang, 1980; Zhu, 1996 Xishancun and Xitun Formations. Lochkovian, 419.2–410.8 Ma. Parayunnanolepis xitunensis Zhang et al., 2001; Zhu et al., 2012 Xitun Formation. Late Lochkovian, 413.6–410.8 Ma. Microbrachius dicki Hemmings, 1978; Long et al., 2015 Eday Flagstone and John O’Groats Sandstone. Lower–middle Givetian, 387.7–384.4 Ma. *Bothriolepis* sp Gogo Young, 1984 Gogo Formation. Early Frasnian, 382.7–379.2 Ma. Bothriolepis canadensis Downs and Donoghue, 2009; Béchard et al., 2014 Escuminac Formation. Early Frasnian, 382.7–379.2 Ma. Pterichthyodes milleri Hemmings, 1978 Achanarras Horizon. Late Eifelian, 389.6–387.7 Ma. Remigolepis walkeri Johanson, 1997 Canowindra. Famennian, 372.2–358.9 Ma. Diandongpetalichthys liaojiaoshanensis Zhu, 1991 Xishancun Formation. Lochkovian, 419.2–410.8 Ma. Quasipetalichthys haikouensis Liu, 1991 Shixiagou Formation, Ninxia. Givetian, 387.7–382.7 Ma. Eurycaraspis incilis (Liu, 1991) Haikou Formation. Givetian, 387.7–382.7 Ma. Lunaspis broili Gross, 1961 Hunsrueck Slate. Late Pragian–early Emsian, 408.7–402.8 Ma. Shearsbyaspis oepiki Young, 1985; Castiello and Brazeau, 2018 Taemas-Wee Jasper. Emsian, 407.6–393.3 Ma. Macropetalichthys rapheidolabis Stensiö, 1925; Stensiö, 1963b; Stensiö, 1969 Onondaga Limetone. Eifelian, 393.3–387.7 Ma. Cowralepis mclachlani Ritchie, 2005; Carr et al., 2009 Merriganowry Shale. Late Givetian–early Frasnian, 384.4–379.2 Ma. Sigaspis lepidophora Goujet, 1973 Wood Bay Formation. Early Pragian, 410.8–409.7 Ma. Kujdanowiaspis podolica Stensiö, 1963a; Dupret, 2010 Dnister Series, Podolia. Late Lockhovian–lower Pragian, 413.6–409.7 Ma. Lehmanosteus hyperboreus Goujet, 1984a Wood Bay Formation. Early Pragian, 410.8–409.7 Ma. Dicksonosteus arcticus Goujet, 1975; Goujet, 1984b Wood Bay Formation. Early Pragian, 410.8–409.7 Ma. *Groenlandaspis* sp Mt Howitt Specimens listed in King et al., 2017 Mt. Howitt. Givetian, 387.7–382.7 Ma. Buchanosteus confertituberculatus Burrow and Turner, 1998; Long et al., 2014 Buchan. Middle–late Pragian, 409.7–407.6 Ma. Parabuchanosteus murrumbidgeensis White and Toombs, 1972; Young, 1979; Burrow and Turner, 1998 Taemas-Wee Jasper. Emsian, 407.6–393.3 Ma. Holonema westolli Miles, 1971 Gogo Formation. Early Frasnian, 382.7–379.2 Ma. Coccosteus cuspidatus Miles and Westoll, 1968 Achanarras and Edderton fish bed. Eifelian-Givetian boundary, 394.5–392.1 Ma. Incisoscutum ritchiei Dennis and Miles, 1981; Giles et al., 2013 Gogo Formation. Early Frasnian, 382.7–379.2 Ma. Eastmanosteus calliaspis Dennis-Bryan, 1987 Gogo Formation. Early Frasnian, 382.7–379.2 Ma. Compagopiscis croucheri Gardiner and Miles, 1994 Gogo Formation. Early Frasnian, 382.7–379.2 Ma. Materpiscis attenboroughi Long et al., 2008; Trinajstic et al., 2012 Gogo Formation. Early Frasnian, 382.7–379.2 Ma. Austroptyctodus gardineri Long, 1997 Gogo Formation. Early Frasnian, 382.7–379.2 Ma. Campbellodus decipiens Long, 1997 Gogo Formation. Early Frasnian, 382.7–379.2 Ma. Rhamphodopsis threiplandi Miles, 1967; (Long, 1997) Edderton Fish Beds. Eifelian-Givetian boundary, 394.5–392.1 Ma. Brindabellaspis stensioi Young, 1980; King et al., 2018 Taemas-Wee Jasper. Emsian, 407.6–393.3 Ma. Romundina stellina Ørvig, 1975; Dupret et al., 2014; Dupret et al., 2017 Prince of Wales Island. Lochkovian, 419.2–410.8 Ma. Jagorina pandora Stensiö, 1969; Young, 1986 Kellwasserkalk, Bad Wildungen. Late Frasnian, 375.7–372.2 Ma. Gemuendina stuertzi Gross, 1963 Hunsrueck Slate. Late Pragian–early Emsian, 408.7–402.8 Ma. Entelognathus primordialis Zhu et al., 2013 Kuanti Formation. Ludlow, 427.4–423 Ma. Qilinyu rostrata Zhu et al., 2016 Kuanti Formation. Ludlow, 427.4–423 Ma. Janusiscus schultzei Giles et al., 2015c Lower Member, Kureika Formation. Middle Lockhovian, 416.4–413.6 Ma. Nerepisacanthus denisoni Burrow, 2011; Burrow and Rudkin, 2014 Bertie Formation. Ludlow–Pridoli, 427.4–419.2 Ma. Poracanthodes menneri Valiukevicius, 1992 Severnaya Zemlya Formation. Early Lockhovian, 419.2–416.4 Ma. Ischnacanthus gracilis Watson, 1937; Miles, 1973a; Burrow et al., 2018 ‘Turin Hill’. Lochkovian, 419.2–410.8 Ma. Tetanopsyrus lindoei/breviacanthias Gagnier et al., 1999; Hanke et al., 2001 MOTH. Lochkovian, 419.2–410.8 Ma. Diplacanthus crassisimus Watson, 1937; Miles, 1973a; Burrow et al., 2016 Moray Firth and Achanarras. Eifelian-Givetian boundary, 394.5–392.1 Ma. Milesacanthus antarctica Young and Burrow, 2004 Aztec Siltstone. Givetian, 387.7–382.7 Ma. Culmacanthus stewarti Long, 1983 Mt Howitt. Givetian, 387.7–382.7 Ma. Euthacanthus macnicoli Watson, 1937; Miles, 1973a; Newman et al., 2014 ‘Turin Hill’. Lockhovian, 419.2–410.8 Ma. Cassidiceps vermiculatus Gagnier and Wilson, 1996 MOTH. Lockhovian, 419.2–410.8 Ma. Promesacanthus eppleri Hanke, 2008 MOTH. Lochkovian, 419.2–410.8 Ma. Mesacanthus mitchelli Watson, 1937; Miles, 1973a ‘Turin Hill’ and Farnell. Lochkovian, 419.2–410.8 Ma. *Cheiracanthus* sp Watson, 1937; Miles, 1973a Middle Old Red Sandstone, Moray Firth. Nodular Fish Beds. Eifelian–Givetian, 393.3–382.7 Ma. Homalacanthus concinnus Gagnier, 1996 Escuminac Formation. Early Frasnian, 382.7–379.2 Ma. Acanthodes bronni Gross, 1935; Watson, 1937; Miles, 1973a; Miles, 1973b; Coates, 1994; Davis et al., 2012; Brazeau and de Winter, 2015 Lebach iIronstone. Asselian, 298.9–293.5 Ma. Ptomacanthus anglicus Miles, 1973a; Brazeau, 2009; Brazeau, 2012; Dearden et al., 2019 Wayne Herbert Quarry. Lochkovian, 419.2–410.8 Ma. Climatius reticulatus Watson, 1937; Miles, 1973a; Burrow et al., 2015 ‘Turin Hill’. Lockhovian, 419.2–410.8 Ma. Vernicomacanthus waynensis Miles, 1973a Wayne Herbert Quarry. Lochkovian, 419.2–410.8 Ma. Parexus recurvus Watson, 1937; Miles, 1973a; Burrow et al., 2013 ‘Turin Hill’. Lochkovian, 419.2–410.8 Ma. Latviacanthus ventspilsensis Schultze and Zidek, 1982 Ventspils. Kemeri stage. Pragian, 410.8–407.6 Ma. Brachyacanthus scutiger Watson, 1937 Lower Old Red Sandstone, Farnell. Lockhovian, 419.2–410.8 Ma. Brochoadmones milesi Hanke and Wilson, 2006 MOTH. Lockhovian, 419.2–410.8 Ma. Gladiobranchus probaton Hanke and Davis, 2008 MOTH. Lochkovian, 419.2–410.8 Ma. Kathemacanthus rosulentus Gagnier and Wilson, 1996; Hanke and Wilson, 2010 MOTH. Lochkovian, 419.2–410.8 Ma. Lupopsyrus pygmaeus Hanke and Davis, 2012 MOTH. Lochkovian, 419.2–410.8 Ma. Obtusacanthus corroconis Hanke and Wilson, 2004 MOTH. Lochkovian, 419.2–410.8 Ma. Gladbachus adentatus Coates et al., 2018 Lower Plattenkalk. Late Givetian, 382.7–384.4 Ma. Cladodoides wildungensis Maisey, 2005 Wildungen Limestone. Late Frasnian, 375.7–372.2 Ma. Akmonistion zangerli Coates and Sequeira, 1998; Coates et al., 1998; Coates and Sequeira, 2001 Manse Burn Formation, Bearsden. Serpukhovian, 330.9–323.2 Ma. *Cobelodus* braincase Maisey, 2007 Fayetteville Formation. Chesterian, 333–318.1 Ma. Cladoselache kepleri/fyleri Harris, 1938; Bendix-Almgreen, 1975; Schaeffer, 1981; Maisey, 2007 Cleveland Member of Ohio Shale. Late Famennian, 363.3–358.9 Ma. Chondrenchelys problematica Moy-Thomas, 1935; Finarelli and Coates, 2011; Finarelli and Coates, 2014 Glencartholm Volcanic Beds. Holkerian, 339–337.5 Ma. Helodus simplex Moy-Thomas, 1936 Fenton, Staffordshire. Moscovian, 315.2–307 Ma. Debeerius ellefseni Grogan and Lund, 2000 Bear Gulch Limestone. Upper Chesterian, 323.1–318.1 Ma. Doliodus latispinosus Miller et al., 2003; Maisey et al., 2009; Maisey et al., 2014; Maisey et al., 2017; Maisey et al., 2018 ‘Atholville Beds’, Campbellton Formation. Emsian–Eifelian, 407.6–391.4 Ma. Hamiltonichthys mapesi Maisey, 1989 Hartford Limetone, Hamilton Quarry. Middle Virgilian, 303.7–298.9 Ma. Onychoselache traquari Dick and Maisey, 1980; Coates and Gess, 2007 Glencartholm Volcanic Beds and Wardie Shales. Holkerian-Asbian, 339–333 Ma. *Orthacanthus* sp Schaeffer, 1981 Admiral fFormation. Wolfcampian, 299–280 Ma. Pucapampella rodrigae Maisey, 2001; Maisey et al., 2018 Sica Sica Formation. Eifelian–Givetian, 393.3–382.7 Ma. Tamiobatis vetustus Schaeffer, 1981; Williams, 1998 Cleveland Shale and Salem lLimestone. Famennian, Early Visean, 372.2–358.9 Ma. Tristychius arcuatus Dick, 1978; Coates and Gess, 2007; Coates and Tietjen, 2018 Wardie Shales and Manse Burn Formation, Bearsden. Late Visean–lower Serpukhovian, 336.2–328.3 Ma. Dialipina salgueiroensis Schultze, 1968; Schultze and Cumbaa, 2001 Bear Rock Formation. Emsian, 407.6–393.3 Ma. *Ligulalepis* braincase Basden et al., 2000; Basden and Young, 2001; Clement et al., 2018 Taemas-Wee Jasper. Emsian, 407.6–393.3 Ma. Cheirolepis canadensis Pearson and Westoll, 1979; Arratia and Cloutier, 1996 Escuminac Formation. Early Frasnian, 382.7–379.2 Ma. Cheirolepis trailli Pearson and Westoll, 1979; Giles et al., 2015a Achanarras Limestone, Tynet Burn and Gamrie. Late Eifelian, 389.6–387.7 Ma. Howqualepis rostridens Long, 1988 Mt. Howitt. Givetian, 387.7–382.7 Ma. Raynerius splendens Giles et al., 2015b Upper part of the Grey Member, Ferques Formation. Conodont zone, 373.5–372.5 Ma. Mimipiscis toombsi Gardiner and Bartram, 1977; Gardiner, 1984; Giles and Friedman, 2014 Gogo Formation. Early Frasnian, 382.7–379.2 Ma. Moythomasia durgaringa Gardiner, 1984 Gogo Formation. Early Frasnian, 382.7–379.2 Ma. Kentuckia deani Rayner, 1952; Giles and Friedman, 2014 New Providence Shale Member, Stockdale Formation. Tournasian-Visean boundary, 347.1–346.3 Ma. Meemannia eos Zhu et al., 2006; Zhu et al., 2010; Lu et al., 2016a Xitun Formation. Late Lochkovian, 413.6–410.8 Ma. Guiyu oneiros Zhu et al., 2009; Qiao and Zhu, 2010 Psarolepis romeri Yu, 1998; Zhu et al., 1999; Zhu and Yu, 2004; Zhu and Yu, 2009 Xishancun Formation. Lochkovian, 419.2–410.8 Ma. Achoania jarvikii Zhu et al., 2001; Zhu and Ahlberg, 2004; Zhu and Yu, 2009 Xitun Formation. Late Lochkovian, 413.6–410.8 Ma. Qingmenodus yui Lu and Zhu, 2009; Lu et al., 2016b Posongchong Formation, Wenshan. Pragian, 410.8–407.6 Ma. Onychodus jandemarrai Andrews et al., 2005 Gogo Formation, Saddler Formation. Early Frasnian, 382.7–379.2 Ma. Miguashaia bureaui Cloutier, 1996; Forey, 1998 Escuminac Formation. Early Frasnian, 382.7–379.2 Ma. Diplocercides kayseri Stensiö, 1922; Jarvik, 1980; Forey, 1998 Wildungen Limestone. Late Frasnian, 375.7–372.2 Ma. Styloichthys changae Zhu and Yu, 2002; Friedman, 2007b Xitun Formation. Late Lochkovian, 413.6–410.8 Ma. Youngolepis praecursor Zhang and Yu, 1981; Chang, 1982; Chang, 1991; Chang and Smith, 1992 Xitun Formation. Late Lochkovian, 413.6–410.8 Ma. Powichthys thorsteinssoni Jessen, 1975; Jessen, 1980; Chang and Smith, 1992 Prince of Wales Island. Late Lockhovian–early Pragian, 413.6–409.7 Ma. Diabolepis speratus Chang and Yu, 1984; Smith and Chang, 1990; Chang, 1995 Xitun Formation. Late Lochkovian, 413.6–410.8 Ma. Dipterus valenciennesi Parrington, 1950; White, 1965; Ahlberg and Trewin, 1994; Challands, 2015 Achanarras Limestone. Late Eifelian, 389.6–387.7 Ma. Rhinodipterus kimberleyensis Clement, 2012; Clement and Ahlberg, 2014 Gogo Formation. Early Frasnian, 382.7–379.2 Ma. ‘Chirodipterus’ australis Miles, 1977; Henderson and Challands, 2018 Gogo fFormation. Early Frasnian, 382.7–379.2 Ma. *Porolepis* sp Jarvik, 1972; Clement, 2004 Wood Bay Formation. Early Pragian, 410.8–409.7 Ma. Glyptolepis groenlandica Jarvik, 1972; Ahlberg, 1989 Red Siltstone Member of the Nathorst Fjord group. Late Eifelian–early Givetian, 389.6–386 Ma. Tungsenia paradoxa Lu et al., 2012; Lu et al., 2019 Posongchong Formation, Wenshan. Pragian, 410.8–407.6 Ma. Kenichthys campbelli Chang and Zhu, 1993; Zhu and Ahlberg, 2004 Chuangdong Formation. Late Emsian, 398.1–393.3 Ma. Osteolepis macrolepidotus Westoll, 1936; Thomson, 1965; Jarvik, 1980 Tynet Burn. Late Eifelian, 389.6–387.7 Ma. Gogonasus andrewsae Long, 1985; Long et al., 1997; Long et al., 2006; Holland, 2013; Holland, 2014 Gogo Formation. Early Frasnian, 382.7–379.2 Ma. Eusthenopteron foordi Jarvik, 1980; Porro et al., 2015 Escuminac Formation. Early Frasnian, 382.7–379.2 Ma. Deleted taxa from King et al., 2017 Wuttagoonaspis fletcheri The description of the braincase of *Wuttagoonaspis* in Ritchie, 1973 does not match direct observations of specimens (by B. King). Impressions of neurocranial processes can be seen lateral to the sensory grooves in several specimens in the Australia Museum collections. However, since these observations are on unpublished specimens, for reproducibility we elected to remove the taxon. Gavinaspis convergens This taxon is known only from an incomplete skull roof, on which the sutures are unclear. Because of the inability to code almost all characters, this specimen was removed from the matrix. *Lehmanosteus* was added as an alternative example of an early arthrodire. Ramirosuarezia boliviana This is another taxon for which the vast majority of characters cannot be scored. Although the specimen consists of a braincase, almost all neurocranial features are uncertain, even the position of the optic nerve foramen. The inability to score most characters justifies removal of the taxon a priori. In addition, it is also notable that two of the suggested attributions of *Ramirosuarezia,* a decayed rhenanid braincase, or a holocephalan (Pradel et al., 2009), receive no support in phylogenetic analyses (e.g. Coates et al., 2018). Conversely, an acanthodian identity (deemed ‘unlikely’ by Pradel et al., 2009), receives some support in phylogenetic analysis (Qiao et al., 2016). Osorioichthys marginis Based on direct observations of the holotype specimen (by B. King), many of the characters scored from the description were unclear or could not be verified. An important character that influences the position of *Osorioichthys* is the described separation of the posterior nostril and orbit by dermal bone. However, observation of the specimen reveals that this is either an artifact of breakage, or represents the postnasal wall of the neurocranium. *Raynerius* was added as an alternative early acintopterygian with better quality preservation of many features.

## Appendix 3

Character list Histology 1. Tessellated calcified cartilage: absent (0); present (1). Burrow et al., 2016 c1. 2. Tessellated calcified cartilage: single-layered (0); multi-layered (1). Maisey, 2001, c17. 3. Perichondral bone: present (0); absent (1). Donoghue et al., 2000, c63. 4. Extensive endochondral ossification: absent (0); present (1). Zhu et al., 2001 c202; Brazeau, 2009 c3. 5. Extensive pore canal network: absent (0); present (1). Giles et al., 2015a c8. 6. Three-layered exoskeleton: absent (0); present (1). Donoghue et al., 2000, c71. 7. Cephalic dermoskeleton bone: cellular (0); acellular (1). Donoghue et al., 2000 c67; Sansom, 2009 c73. 8. Perforated horizontal lamina in the sensory canals and vascular system: absent (0); present (1). Sansom, 2009 c85. 9. Galeaspidin: absent (0); present (1). Sansom, 2009 c87. 10. Dentine: absent (0); present (1). Brazeau, 2009 c4. 11. Dentine kind: mesodentine (0); semidentine (1); orthodentine (2). Brazeau, 2009 c5. 12. Bone cell lacunae in body scale bases: present (0); absent (1). Burrow and Turner, 2010 c61. 13. Main dentinous tissue forming fin spine: osteodentine (0); orthodentine (1). Burrow and Turner, 2010 c60. 14. Resorption and redeposition of odontodes: absent or partially developed (0); present (1). Zhu et al., 2006 c122. 15. Enamel(oid) present on dermal bones and scales: absent (0); present (1). Giles et al., 2015b c5. 16. Enamel: single-layered (0); multi-layered (1). Giles et al., 2015a c6. 17. Enamel layers: applied directly to one another (ganoine) (0); separated by layers of dentine (1). Giles et al., 2015b c7. Braincase proportions 18. Nasal opening(s): dorsal, placed between orbits (0); ventral and anterior to orbits (1). Friedman, 2007a c142. 19. Nasal capsules in anterlolateral corners of orbit: no (0); yes (1). King et al., 2017 c96. 20. Elongate preorbital region between orbits and nasal capsules: absent (0); present (1). King et al., 2017 c22. 21. Ethmoid region elongate with dorsoventrally deep lateral walls: absent (0); present (1). Davis et al., 2012 c73. 22. Tectum orbitale/supraorbital shelf: absent or very narrow (0); present (1). Ahlberg and Johanson, 1998b c83; King et al., 2017 c102. 23. Supraorbital shelf broad with convex lateral margin: absent (0); present (1). Coates and Sequeira, 1998 c17. 24. Orbit dorsal or facing dorsolaterally, surrounded laterally by endocranium: present (0); absent (1). Brazeau, 2009 c68. 25. Narrow interorbital septum: absent (0); present (1). Brazeau, 2009 c70. 26. Extended prehypophysial portion of sphenoid: absent (0); present (1). Brazeau, 2009 c69. 27. Short otico-occipital region of braincase: absent (0); present (1). Brazeau, 2009 c74. 28. Parachordal shape: broad, flat (0); keeled with sloping lateral margins (1). Brazeau, 2009 c98. 29. Stalk-shaped parachordal/occipital region: absent (0); present (1). Giles et al., 2015a c176. 30. Braincase is series of bilateral ossifications: no (0); yes (1). King et al., 2017 c100. Braincase processes 31. Median rostral dorsal process of braincase: absent (0); present (1). King et al., 2017 c101. 32. Rostral processes: absent (0); present (1). King et al., 2017 c99. 33. Postnasal wall: absent (0); present (1). Clement et al., 2018 c281. 34. Processus supraorbitalis lateralis: absent (0); present (1). King et al., 2017 c110. 35. Postorbital process: absent (0); present (1). Giles et al., 2015b c132. 36. Transverse otic process: present (0); absent (1). Giles et al., 2015c c125. 37. Transverse otic process: not extending in front of orbits (0); extending in front of orbits (1). Jia et al., 2010 c95. 38. Branchial ridges: present (0); reduced to vagal process (1); absent (articulation made with bare cranial wall) (2). Giles et al., 2015c c166. 39. Vagal process: forked (0); unforked (1). Pan et al., 2015 c14; King et al., 2017 c97. 40. Craniospinal process: absent (0); present (1). Giles et al., 2015a c167. 41. Paravagal fossa: absent (0); present (1). Pan et al., 2015 c18. Braincase fontanelles and fissures 42. Optic fissure: present (0); absent (1). Dupret et al., 2014 c255. 43. Ventral cranial fissure: absent (0); present (1). Brazeau, 2009 c92. 44. Anterior margin of ventral fissure: straight (0); sinusoidal (1). King et al., 2017 c127. 45. Endoskeletal cranial joint: absent (0); present (1). Brazeau, 2009 c64. 46. Ventral notch between parachordals: absent (0); present or entirely unfused (1). Brazeau, 2009 c97. 47. Parachordal plates: separated from the otic capsule (0); sutured or fused to otic capsule (1). Friedman, 2007a c182. 48. Metotic (otic-occipital) fissure: absent (0); present (1). Brazeau, 2009 c93. 49. Occipital arch wedged in between otic capsules: absent (0); present (1). Coates and Sequeira, 1998 c9. 50. Precerebral fontanelle: absent (0); present (1). Coates and Sequeira, 1998 c21. 51. Anterolateral fenestra in roof of otoccipital: absent (0); present (1). King et al., 2017 c111. 52. Hypophysial foramen in braincase: absent (0); present (1). King et al., 2017 c114. 53. Posterior dorsal fontanelle: absent (0); present (1). Brazeau, 2009 c85. 54. Shape of posterior dorsal fontanelle: approximately as long as broad (0); much longer than wide, slot-shaped (1). Coates and Sequeira, 1998 c10. 55. Perilymphatic fenestra: absent (0); present (1). Pradel et al., 2011 c16. 56. Vestibular fontanelle: absent (0); present (1). Friedman, 2007b c180. 57. Ventral cranial fissure connects with vestibular fontanelles: no (0); yes (1). Coates, 1999 c29; King et al., 2017 c112. 58. Basal fenestra opening into floor of orbit: absent (0); present (1). King et al., 2017 c129. 59. Subpituitary fenestra: absent (0); present (1). Goujet and Young, 1995 c12. 60. Basicranial fenestra: absent (0); present (1). Ahlberg and Johanson, 1998b c76. Myodomes and articulations 61. Vomeral area with grooves and raised areas: absent (0); present (1). Zhu and Schultze, 2001 c142. 62. Ethmoidal articulation of palatoquadrate: absent (0); present (1). King et al., 2017 c122. 63. Ethmoid articulation for palatoquadrate: extends posteriorly to the level of N.II (0); placed on postnasal wall (1); majority of facet anterior to postnasal wall (2). Friedman, 2007a c172. 64. Eye stalk or unfinished area on neurocranial wall for eye stalk: absent (0); present (1). Zhu and Schultze, 2001 c36. 65. Position of basal/basipterygoid articulation: same anteroposterior level as hypophysial opening (0); anterior to hypophysial opening (1). Brazeau, 2009 c81. 66. Basipterygoid process (basal articulation) with vertically oriented component: absent (0); present (1). Brazeau, 2009 c83. 67. Expanded articular area anterior to basipterygoid process: absent (0); present (1). King et al., 2017 c103. 68. Orbital/palatobasal articulation: posterior to optic foramen (0); anterior to optic foramen (1). King et al., 2017 c123. 69. Descending process of sphenoid (with its posterior extremity lacking periostegeal lining): absent (0); present (1). Ahlberg, 1991 c53. 70. Processus connectens: short (0); elongate (1). Lu et al., 2016a c66. 71. Articulation between neurocanium and palatoquadrate posterodorsal to orbit (suprapterygoid articulation): absent (0); present (1). Giles et al., 2015a c147. 72. Division of postorbital palatoquadrate articulation into dorsal and ventral components: absent (0); present (1). New, adapted from King et al., 2017 c128. Two condyles in the dorsal otic region are found in *Acanthodes* and *Homalacanthus*. 73. Periotic process: absent (0); present (1). Pradel et al., 2011 c11. 74. Hyomandibula articulating with braincase: yes (0); no (1). King et al., 2017 c121. 75. Position of hyomandibular articulation on neurocranium: Anterior position, suborbital (0); posterior position, behind orbit (1). Brazeau, 2009 c89; King et al., 2017 c369. 76. Relative position of jugular groove and hyomandibular articulation: hyomandibula dorsal or same level (0); jugular vein passing dorsal or lateral to hyomandibula (1). Brazeau and de Winter, 2015 c237. 77. Articulation facet with hyomandibular: single-headed (0); double-headed (1). Zhu and Schultze, 2001 c128. 78. Hyomandibular facets where they straddle the jugular canal: narrowly separated (0); broadly separated (1). Friedman, 2007b c176. 79. Articulation surface on ventrolateral surface of otic capsule: absent (0); present (1). New. There is some uncertainty what articulation surfaces on the otic capsule wall are for (see e.g. Chang, 1982), but they are most commonly assumed to be for the articulation of an infrapharyngobranchial. This character simply codes the presence or absence of an articulation. 80. Paired occipital facets: absent (0); present (1). Giles et al., 2015b c177. 81. Position of myodome for superior oblique eye muscles: posterior and dorsal to foramen for nerve II (0); anterior and dorsal to foramen for nerve II (1). Brazeau, 2009 c63. 82. Medial recess of the posteroventral myodome: absent (0); present (1). Sansom, 2009 c103. Braincase ridges 83. Pronounced sub-ethmoidal keel: absent (0); present (1). Coates and Sequeira, 1998 c22; Brazeau, 2009 c62. 84. Subcranial ridges: absent (0); present (1). Giles et al., 2015a c141. 85. Dorsal ridge: absent (0); present (1). Coates and Sequeira, 1998 c11; Davis et al., 2012 c91. Includes the unpaired median crista of lungfishes. 86. Shape of median dorsal ridge anterior to endolymphatic fossa: developed as a squared-off ridge or otherwise ungrooved (0); bears a midline groove (1). Giles et al., 2015a c161. 87. Dorsal otic ridge forming a horizontal crest: absent (0); present (1). Coates and Sequeira, 1998 c11; Pradel et al., 2011 c12. 88. Hypotic lamina (and dorsally directed glossopharyngeal canal): absent (0); present (1). Davis et al., 2012 c103. 89. Dorsolateral cristae: absent (0); present (1). New. 90. Dorsolateral cristae fenestrated: no (0); yes (1). Friedman, 2007a c16. 91. Lateral (parotic) cristae: absent (0); present (1). New. The parotic crista of sarcopterygians and the lateral cristae of lungfishes are here considered potential homologues following Miles, 1977. Friedman, 2007b compares the dorsolateral cristae as homologous to the parotic cristae, but cites Miles, so this is assumed to be erroneous. Notochord 92. Size of aperture to notochordal canal: much smaller than foramen magnum (0); as large, or larger, than foramen magnum (1). Giles et al., 2015a c178. 93. Notochord short and stopped by occipital cotylus: absent (0); present (1). Pradel et al., 2011 c21. 94. Unconstricted cranial notochord: absent (0); present (1). Ahlberg and Johanson, 1998b c153. Spiracle 95. Spiracular groove on basicranial surface: absent (0); present (1). Brazeau, 2009 c65. 96. Spiracular groove on lateral commissure: absent (0); present (1). Davis et al., 2012 c63. 97. Endoskeletal spiracular enclosure: absent (0); present (1); spiracular canal (2). Clement et al., 2018 c267. Endocast 98. Relationship of cranial endocavity to basisphenoid: endocavity occupies full depth of sphenoid (0); endocavity dorsally restricted (1). Giles et al., 2015b c140. 99. Nasal sacs: unpaired (0); paired (1). King et al., 2017 c130. 100. Olfactory tracts: separate tracts (0); single anterior division of the cranial cavity (1). New. In coelacanths there are no separate canals for the olfactory nerves but rather a large anterior division of the endocavity through which the olfactory tracts pass. 101. Rostral organ: absent (0); present (1). Friedman, 2007b c145. 102. Hypophyseal chamber: projects posteroventrally (0); projects ventrally or anteroventrally (1). Clement et al., 2018 c270. 103. Paired recesses anterior of hypophysial fossa: absent (0); present, blind (1); present, connect via canals to cranial cavity (2). New. *Tungsenia* has paired recesses at the anterior of the hypophysial recess (Lu et al., 2012 fig. 4b ?PT), interpreted as similar to the pars tuberalis of living urodeles. In *Glyptolepis* there are also extensions of the hypophysial recess that connect anteriorly to the cranial cavity (Jarvik, 1972 fig. 17A c.p.tub). 104. Optic lobes: narrower than cerebellum (0); same width or wider than cerebellum (1). Lu et al., 2017 c271. 105. Lateral cranial canal: absent (0); present (1). Coates, 1999 c32. 106. Supraotic cavity: absent (0); present (1). Lu et al., 2017 c275. 107. Endolymphatic ducts: posteriodorsally angled tubes (0); tubes oriented vertically through median endolymphatic fossa (1). Coates and Sequeira, 2001 c73; Brazeau, 2009 c87. Inner ear 108. Labyrinth cavity: separated from the main neurocranial cavity by a cartilaginous or ossified capsular wall (0); skeletal capsular wall absent (1). Davis et al., 2012 c82. 109. External (horizontal) semicircular canal: absent (0); present (1). Brazeau, 2009 c83. 110. External (horizontal) semicircular canal: joins the vestibular region dorsal to posterior ampulla (0); joins level with posterior ampulla (1). Davis et al., 2012 c87. 111. Horizontal semicircular canal in dorsal view: medial to path of jugular vein (0); dorsal to jugular vein (1). Giles et al., 2015b c154. 112. Horizontal semicircular canal: horizontally orientated (0); obliquely orientated (1). Lu et al., 2017 c274. 113. Crus commune: dorsal to endocranial roof (0); ventral to endocranial roof (1). Lu et al., 2017 c272. 114. Sinus superior: absent or indistinguishable from union of anterior and posterior canals with saccular chamber (0); present (1). Davis et al., 2012 c86. 115. Utricular recess: absent (0); present small (1); present large (2). New. A diverticulum of the labyrinth cavity at the junction of the external semicircular canal and the sacculus, interpreted as housing the utriculus. See Brazeau and Friedman, 2014 p18 for discussion. 116. Lagenar recess: absent (0); present (1). New. A large recess at the posterior end of the saccular chamber for the lagena is well developed in *Diplocercides* (Jarvik, 1980, fig.217 space for lagena). Recently, a similar recess was described for the chondrichthyan *Tristychius* (Coates et al., 2018, fig.11). 117. Number of SEL canals: five (0); less than 5 (1). Sansom, 2009 c91. 118. SEL one canal bifurcation: between orbit and lateral field (0); close to field (1); close to orbit (2). Sansom, 2009 c110; King et al., 2017 c92. Blood vessels 119. Canal for efferent pseudobranchial artery within basicranial cartilage: absent (0); present (1). Brazeau, 2009 c80. 120. Entrance of internal carotids in ‘tropibasic’ braincase: through basisphenoid pillar (0); through orbits (1). New. Revised from King et al., 2017 c38 and c125 to remove redundancy. This character is only applicable to taxa with a 'tropibasic' braincase: i.e. it is dependent on character 98. 121. Entrance of internal carotids: through separate openings flanking the hypophyseal opening or recess (0); through a common opening at the central midline of the basicranium (1). Brazeau, 2009 c79. 122. Canal for lateral dorsal aorta within basicranial cartilage: absent (0); present (1). Coates and Sequeira, 1998 c4; Brazeau, 2009 c78. 123. Midline canal in basicranium for dorsal aorta: absent (0); present (1). Zhu et al., 2013 c234. 124. Jugular canal: long (invested in otic region along length of skeletal labyrinth) (0); short (restricted to region anterior of skeletal labyrinth) (1); absent (jugular vein uninvested in otic region) (2). Giles et al., 2015b c126. 125. Canal for jugular in postorbital process: absent (0); present (1). Giles et al., 2015c c133. 126. Pituitary vein canal: dorsal to level of basipterygoid process (0); flanked posteriorly by basipterygoid process (1). Davis et al., 2012 c84. 127. Pituitary vein canal: Discontinuous, enters cranial cavity (0); Discontinuous, enters hypophysial recess (1); Continuous transverse canal (2). Clement et al., 2018 c282. 128. Marginal vein: absent (0); present (1). Sansom, 2009 c93. Cranial nerves 129. Olfactory tracts: short, with olfactory capsules situated close to telencephalon cavity (0); elongate and tubular (much longer than wide) (1). Brazeau, 2009 c60. 130. Rostral tubuli: absent (0); present (1). Cloutier and Ahlberg, 1996 c77. 131. Profundus and trigeminal nerves: emerge from cranial cavity separately (0); emerge from cranial cavity together (1). King et al., 2017 c94. 132. Size of anterior profundus canal: small (0); large (1). Zhu and Schultze, 2001 c144 Definition revised to remove the postnasal wall, as an anterior profundus foramen can sill be present when the posnasal wall is poorly developed, in particular in ‘*Ligulalepis’*. 133. Series of perforations for innervation of supraorbital sensory canal in supraorbital shelf: absent (0); present (1). Giles et al., 2015c c134. 134. Profundus nerve enters orbit with jugular vein: no (0); yes (1). New. In general, the profundus nerve enters the orbit through a foramen on the posteriodorsal wall. However, in *'Chirodipterus'* the profundus nerve first joins the jugular vein canal (Henderson and Challands, 2018 p16). 135. Relative position of trigeminal nerve: behind endoskeletal cranial joint (0); through endoskeletal cranial joint (1); anterior to endoskeletal joint (2). Zhu and Schultze, 2001 c134. This character is here adapted to include an extra state in when the intracranial joint is behind the trigeminal nerve. It is dependent on the presence of an intracranial joint. 136. Palatine nerve canal: absent (0); present (1). New. A palatine nerve canal is present in gnathostomes, but is apparently unknown in osteostracans or galeaspids (see discussion in King et al., 2017) 137. Hyoid ramus of facial nerve (N. VII) exits through posterior jugular opening: absent (0); present (1). Friedman, 2007b c179. 138. Glossopharyngeal nerve (N. IX) exit: foramen situated posteroventral to otic capsule and anterior to metotic fissure (0); through metotic fissure (1). Coates and Sequeira, 1998 c2; Brazeau, 2009 c73. 139. Spino-occipital nerve foramina: two or more, aligned horizontally (0); one or two, dorsoventrally offset (1). Coates and Sequeira, 1998 c8; Brazeau, 2009 c95. Dermal palate bones 140. Median dermal bone of palate (parasphenoid): absent (0); present (1). Brazeau, 2009 c57. 141. Ascending process of parasphenoid: absent (0); present (1). Patterson, 1982a c9. 142. Shape of parasphenoid denticulated field: broad rhomboid or lozenge-shaped (0); broad, splint-shaped (1); slender, splint-shaped (2). Friedman, 2007b c168. 143. Parasphenoid denticulated field with multifid anterior margin: absent (0); present (1). Friedman, 2007b c167. 144. Parasphenoid: protruding forward into ethmoid region of endocranium (0); behind ethmoid region (1). Zhu and Schultze, 2001 c124. 145. Denticulated field of parasphenoid: without spiracular groove (0); with spiracular groove (1). Friedman, 2007b c82. 146. Parasphenoid denticle field: terminates at or anterior to level of foramina for internal carotid arteries (0); extends posterior to foramina for internal carotid arteries (1). Friedman, 2007b c170. 147. Four carotid foramina in parasphenoid: absent (0); present (1). Lu et al., 2012 c98; King et al., 2017 c138. 148. Buccohypophysial canal in parasphenoid: single (0); paired (1). Giles et al., 2015a c114. 149. Posterior stalk of parasphenoid covering otic region: absent (0); present (1). Friedman, 2007a c63. 150. Parasphenoid posterior stalk furrow: absent (0); present (1). Schultze, 2001 c51. 151. Prespiracular dental plate: absent (0); present (1). King et al., 2017 c108. 152. Parotic dental plate: absent (0); present (1). King et al., 2017 c139. Skull roof, overall features 153. Dermal skull roof: includes large dermal plates (0); consists of undifferentiated plates or tesserae (1). Brazeau, 2009 c19. 154. Tesserae morphology: large interlocking polygonal plates (0); microsquamose, not larger than body tesserae (1). Brazeau, 2009 c20. 155. Extent of dermatocranial cover: complete (0); incomplete (scale-free cheek and elsewhere) (1). Brazeau, 2009 c21. 156. Series of paired median skull roofing bones that meet at the dorsal midline of the skull (rectilinear skull roof pattern): absent (0); present (1). Brazeau, 2009 c24. 157. Dermal intracranial joint: absent (0); present (1). Cloutier and Ahlberg, 1996 c81. 158. Cranial spines: absent (0); present, multicuspid (1); present, monocuspid (2). Giles et al., 2015c c36. Skull roof, foramina 159. Pineal opening perforation in dermal skull roof: absent (0); present (1). Brazeau, 2009 c26. 160. Location of pineal foramen/eminence: level with posterior margin of orbits (0); well posterior of orbits (1). Ahlberg and Johanson, 1998b c37. 161. Endolymphatic ducts open in dermal skull roof: present (0); absent (1). Brazeau, 2009 c22. 162. Endolymphatic ducts with oblique course through dermal skull bones: absent (0); present (1). Goujet and Young, 1995 c8. 163. Endolymphatic duct relationship to median skull roof bone (i.e. nuchal plate): within median bone (0); on bones flanking the median bone (e.g. paranuchals) (1). Giles et al., 2015c c40. 164. Dermal plate associated with pineal eminence or foramen: contributes to orbital margin (0); plate bordered laterally by skull roofing bones (1). Giles et al., 2015c c42. Skull roof, snout 165. Median rostral extension of head shield: absent (0); present (1). Sansom, 2009 c1. 166. Tooth-bearing median rostral: absent (0); present (1). Cloutier and Ahlberg, 1996 c22. 167. T-shaped rostral: absent (0); present (1). Carr and Hlavin, 2010 c5; King et al., 2017 c237. 168. Multiple postrostral bones: no (0); yes (1). New. Homology of snout bones (i.e. the bones anterior to the parietals) across gnathostomes are difficult to assess. This character simply makes the distinction between the mosaic of small irregular bones (postrostrals, nasals, tectals) found in sarcopterygians with the relatively small number of larger plates in actinopterygians and placoderms. 169. Number of median bones anterior to parietals: none (0); one (1); two (2). New. This character reformulates a number of previous characters regarding the presence of rostral and premedian plates. In placoderms the first median bone anterior to the patietals (preorbitals) is generally termed the rostral, while the second is called the premedian or internasal. In osteichthyans they are termed as the postrostral and rostral. Here we remove the position of the nasal capsules from the definition of a premedian plate (e.g. Zhu et al. c148) as the position of the nasal capsules is dealt with in other characters. In taxa with a rostral mosaic of bones (character 168), this character is considered inapplicable. 170. Premedian plate: large plate (0); reduced to internasal plate (1). Zhu et al., 2016 c157, revised. This character is contingent on the presence of a premedian plate. This is covered in character 169 state 2. 171. Paired prenostril trenches on premedian plate: absent (0); present (1). New. Paired prenostril trenches are present on the premedian/internasal plate of Qilinyu. This character is contingent on the presence of a premedian plate (Character 169 state 2). 172. Unornamented shelf and rostrocaudal groove on premedian: absent (0); present (1). Jia et al., 2010 c3. 173. Preorbital depression: absent (0); present (1). Jia et al., 2010 c6. 174. Supraorbital: absent (0); present (1). Cloutier and Ahlberg, 1996 c28. 175. Lateral plate: absent (0); present (1). Zhu et al., 2013 c157. 176. Prelateral plate: absent (0); present (1). King et al., 2017 c251. 177. Submarginal articulation: absent (0); present (1). Jia et al., 2010 c16. 178. Parietals (preorbitals of placoderms) surround pineal foramentotoeminence: yes (0); no (1). Ahlberg and Johanson, 1998b c38. 179. Median bone separating parietals: absent (0); present (1); present and separates postparietals as well (2). King et al., 2017 c271, revised. 180. Paraorbital plate separating suborbital from orbit: absent (0); present (1). King et al., 2017 c253. Skull roof, sclerotic ring 181. Sclerotic ring: absent (0); present (1). Giles et al., 2015a c52. 182. Number of sclerotic plates: four or less (0); more than four (1). Cloutier and Ahlberg, 1996 c49. 183. Sclerotic ring incorporated into skull: no (0); yes (1). King et al., 2017 c244. Skull roof, back half 184. Dermal bone (sarcopt postorbital) between jugal and intertemporal: absent (0); present (1). King et al., 2017 c279. 185. Complete enclosure of spiracle by skull roof bones: absent (0); present (1). Friedman, 2007b c148. 186. Suture between paired skull roofing bones (centrals of placoderms postparietals of osteichthyans): straight (0); sinusoidal (1). Giles et al., 2015a c49. 187. Number of bones bearing otic canal between dermosphenotic and lateral extrascapular: one (0); two (1); more than two (2). New. These bones are termed the marginal and anterior paranuchal in placoderms and the supratemporal and tabular in osteichthyans. 188. Supratemporal contact with postparietal: present (0); absent due to anterior displacement (1); absent due to lateral displacement (2). Swartz, 2009 c15; King et al., 2017 c273. 189. Supratemporal contact with nasal: (0); present (1). Gardiner and Schaeffer, 1989 c26. 190. Contact of tabular or anterior paranuchal with postparietal or central: less than half length of postparietal (0); extends most of the length of postparietal (1). New. 191. Series of bones lateral to supratemporal series: absent (0); single bone (1); two bones (2). King et al., 2017 c263. 192. Number of extrascapulars: uneven (0); paired (1). Cloutier and Ahlberg, 1996 c40. 193. Number of paired extrascapulars: one pair (0); two pairs (1). Gardiner and Schaeffer, 1989 cA8. 194. Medial processes of paranuchal wrapping posterolateral corners of nuchal plate: absent (0); present (1); paranuchals precluded from nuchal by centrals (2). Giles et al., 2015a c50. The fourth state of the Giles et al character is not included here (and the taxa are considered inapplicable) as the presence of a median posterior skull roof bone is dealt with by character 192. 195. Nuchal plate: without orbital facets (0); with orbital facets (1). Jia et al., 2010 c14. 196. Centronuchal plate: absent (0); present (1). Dupret et al., 2009 c17. 197. Contact of nuchal or centronuchal plate with paired preorbital plates: absent (0); present (1). Zhu et al., 2013 c164. 198. Postnuchal plate: absent (0); present (1). Dupret et al., 2009 c45; King et al., 2017 c239. 199. Presupracleithrum: absent (0); present (1). Patterson, 1982b c13. 200. Fused scale rows on head shield: absent (0); present (1). Sansom, 2009 c43. 201. Dorsal spinal process on head shield: absent (0); present (1). Sansom, 2009 c44. 202. Cornual extensions: absent (0); present (1). Sansom, 2009 c36; Zhu and Gai, 2007 c14. 203. Spines on cornual extension: absent (0); present (1). Zhu and Gai, 2007 c18. 204. Most posterior bones flanking postparietals: level with posterior margin of postparietals (0); extend posterior to posterior margin of potparietals (1). Lu et al., 2016b c238. Skull roof, joint 205. Type of dermal neck-joint: overlap (0); ginglymoid (1). Zhu et al., 2013 c169; Giles et al., 2015a c60. 206. Type of ginglymoid neck-joint: conventional (0); reverse (1). King et al., 2017 c174. 207. Dermal neck-joint between paired main-lateral-line-bearing bones of skull and shoulder girdle: absent (0); present (1). Zhu et al., 2013 c168. Skull roof, visceral 208. Broad supraorbital vaults: absent (0); present (1). Giles et al., 2015a c44. 209. Paired pits on ventral surface of nuchal plate: absent (0); present (1). Giles et al., 2015b c51. 210. Preorbital recess: absent (0); present (1). Jia et al., 2010 c8; King et al., 2017 c247. 211. Preorbital recess: restricted to premedian plate (0); extends to lateral plates (1). Jia et al., 2010 c8; King et al., 2017 c248. 212. Posterior descending lamina of skull roof: absent (0); present (1). Pan et al., 2015 c6. 213. Mesial lamina of marginal plate: absent (0); present (1). King et al., 2017 c254. Skull roof, fields 214. Lateral fields: absent (0); present (1). Sansom, 2009 c4. 215. Division of lateral fields: absent (0); divided once (1); divided twice (2). Sansom, 2009 c5-6; King et al., 2017 c220. 216. Lateral fields extend posterior to pectoral sinus: absent (0); present (1). Sansom, 2009 c10. 217. Lateral field extends to cornua: no (0); yes (1). Sansom, 2009 c11. 218. Median field: absent (0); present (1). Sansom, 2009 c13. 219. Median field separation from pineal plate or foramen: absent (0); present (1). Sansom, 2009 c15. 220. External endolymphatic duct opens within median field: internal (0); external (1). Sansom, 2009 c17. 221. Median dorsal opening: absent (0); present (1). Donoghue et al., 2000 c14; Zhu and Gai, 2007 c1. 222. Shape of median dorsal opening: transverse slit-like (0); oval-like (1); slender longitudinal oval (2). Zhu and Gai, 2007 c6. Nostrils 223. External nasal opening: single median (0); paired (1). Donoghue et al., 2000 c14; Sansom, 2009 c25. 224. Nostrils enclosed in dermal skull roof: yes (0); no (1). King et al., 2017 c256. 225. Nasohypophysial opening shape: unconstricted (0); constricted (1); split (2). Sansom, 2009 c228; King et al., 2017 c228. 226. Posterior nostril: associated with orbit (0); not associated with orbit (1). Cloutier and Ahlberg, 1996 c46. 227. Dermintermedial process: absent (0); present (1). Zhu and Schultze, 2001 c37. 228. Position of posterior nostril: external, far from jaw margin (0); external, close to jaw margin (1); palatal (2). Zhu and Schultze, 2001 c39. 229. Lacrimal posteriorly enclosing posterior nostril: absent (0); present (1). Zhu and Schultze, 2001 c28. 230. Premaxilla contributes to posterior nostril: absent (0); present (1). Friedman and Blom, 2006 c7. Operculogular 231. Opercular cover of branchial chamber: complete or partial (0); separate gill covers and gill slits (1). Davis et al., 2012 c32. 232. Branchiostegals: absent (0); present (1). Brazeau, 2009 c31. 233. Branchiostegal plate series along ventral margin of lower jaw: absent (0); present (1). Brazeau, 2009 c32. 234. Branchiostegal ossifications: plate-like (0); narrow and ribbon-like (1). Brazeau, 2009 c33; Davis et al., 2012 c29. 235. Branchiostegal ossifications: ornamented (0); unornamented (1). Brazeau, 2009 c34. 236. Imbricated branchiostegal ossifications: absent (0); present (1). Brazeau, 2009 c35. 237. Shape of opercular (submarginal) ossification: broad plate that tapers towards its proximal end (0); narrow, rod-shaped (1). Brazeau, 2009 c37. 238. Size of lateral gular plates: extending most of length of the lower jaw (0); restricted to the anterior third of the jaw (no longer than the width of three or four branchiostegals (1)). Brazeau, 2009 c39. 239. Median gular: present (0); absent (1). Cloutier and Ahlberg, 1996 c66. 240. Number of branchiostegal rays per side: 10 or more (0); 2–7 (1); one (2). Cloutier and Ahlberg, 1996 c63. 241. Accessory operculum: absent (0); present (1). Dietze, 2000 c56. 242. Oralobranchial covering: tesserae (0); plates (1). Sansom, 2009 c60; King et al., 2017 c232. 243. Headshield enclosed posteriorly behind oralobranchial chamber: no (0); yes (1). King et al., 2017 c235. Cheek 244. Cheek plate: undivided (0); divided (i.e. squamosal and preopercular) (1). Giles et al., 2015a c54. 245. Subsquamosals in taxa with divided cheek: absent (0); present (1). Zhu and Schultze, 2001 c64; Giles et al., 2015b c54. 246. Preopercular shape: rhombic (0); bar-shaped (1). Zhu and Schultze, 2001 c71. 247. Consolidated cheek plates: absent (0); present (1). Brazeau, 2009 c25. 248. Enlarged postorbital tessera separate from orbital series: absent (0); present (1). Brazeau, 2009 c30. 249. Most posterior major bone of cheek bearing preopercular canal (preopercular) extending forward, close to orbit: absent (0); present (1). Zhu and Schultze, 2001 c58; Zhu et al., 2009 c59. 250. Contact between most posterior major bone of cheek bearing preopercular canal and maxilla: present (0); absent (1). Zhu and Schultze, 2001 c66. 251. Bone bearing both quadratojugal pit-line and preopercular canal: absent (0); present (1). Friedman, 2007b c42. 252. Notch in anterior margin of jugal: absent (0); present (1). Cloutier and Arratia, 2004 c81. 253. Quadratojugal: present (0); absent (1). Zhu and Schultze, 2001 c57; Dietze, 2000 c31. 254. Lacrimal: absent (0); present (1). King et al., 2017 c257. Premaxilla/maxilla 255. Premaxilla: extends under orbit (0); restricted anterior to orbit (1). Friedman, 2007b c150. 256. Premaxillae with inturned symphysial processes: absent (0); present (1). Friedman, 2007b c149. 257. Premaxilla forming part of orbit: absent (0); present (1). Cloutier and Arratia, 2004 c18. 258. Posterior expansion of maxilla (maxilla cleaver-shaped): present (0); absent (1). Lund et al., 1995 c52; Zhu and Schultze, 2001 c54. 259. Contribution by maxilla to posterior margin of cheek: present (0); absent (1). Friedman, 2007b c151. 260. Ventral margin of maxilla: straight (0); curved (1). Dietze, 2000 c26. 261. Orbital process of maxilla: absent (0); present (1). King et al., 2017 c282. Dermal ornament and pores 262. Dermal ornamentation: smooth (0); ridges (1); tuberculate (2). Giles et al., 2015b c29; King et al., 2017 c205. 263. Size of cosmine pores: small (0); large (1). Zhu et al., 2001 c149. 264. Pore clusters: absent (0); present (1). Zhu and Schultze, 2001 c207. 265. Westoll-lines: absent (0); present (1). Zhu and Schultze, 2001 c207. 266. Transverse external groove behind pineal opening: absent (0); present (1). King et al., 2017 c255. 267. Sensory foramina on skull roof, behind orbits: absent (0); present (1). King et al., 2017 c241. 268. Cutaneous pits on cheek bones: absent (0); present (1). New. Here we combine previous characters dealing with sensory pits on the cheeks of osteichthyans (Ahlberg and Johanson, 1998b 63) and placoderms (King et al., 2017 240, 241). There is no a priori reason to reject homology of these pits. Teeth 269. Oral dermal tubercles borne on jaw cartilages: absent (0); present (1). Brazeau, 2009 c41. 270. Oral tubercles in patterned rows (teeth): absent (0); present (1). Brazeau, 2009 c42. 271. Basal resorption of teeth: absent (0); present (1). New. This character can be scored based on the presence of replacement pits. 272. Oral dermal tubercles fused to jaw cartilages: absent (0); present (1). New. *Pucapampella* has the unusual condition in which statodont teeth are fused directly to the jaw cartilage, which extended into the core of the teeth (Maisey et al., 2018 p99). 273. Tooth whorls: absent (0); present (1). Brazeau, 2009 c43. 274. Tooth whorls extent: at symphysis (0); along entire jaw (1). Giles et al., 2015b c83. 275. Distribution of marginal tooth whorls: upper and lower jaws (0); lower jaws only (1); upper jaws only (2). Giles et al., 2015b c84. 276. Bases of marginal tooth whorls: single, continuous plate (0); some or all whorls consist of separate tooth units (1). Brazeau, 2009 c44; Davis et al., 2012 c41. 277. Toothplates: absent (0); present (1). Coates et al., 2018 c85. 278. Extramandibular dentition: absent (0); present (1). Lu et al., 2012 c392. 279. Number of tooth rows on outer dental arcade: one (0); two (1). Friedman, 2007a c157; King et al., 2017 c380. 280. Teeth of dentary: reaching anterior end of dentary (0); not reaching anterior end (1). Ahlberg and Johanson, 1998b c11. 281. Fangs of coronoids (sensu stricto): absent (0); present (1). Ahlberg et al., 2000 c15. 282. Number of fang pairs on posterior coronoid: one (0); two (1); none (2). Ahlberg and Johanson, 1998b c13. 283. Marginal denticle band on coronoids: broad band, at least posteriorly (0); narrow band with 2–4 denticle rows (1). Ahlberg and Johanson, 1998b c9. 284. Teeth radial rows on prearticular: absent (0); present (1). Zhu and Schultze, 2001 c95. 285. Core of oral dermal tubercles: open pulp cavity (0); vascular network (osteodentine) (1). New. We do not differentiate between the absence of a pulp cavity and a secondarily infilled pulp cavity. 286. Enamel(oid) on teeth: absent (0); present (1). Giles et al., 2015a c79. 287. Plicidentine: absent (0); present (1). Cloutier and Ahlberg, 1996 c86. 288. Acrodin: absent (0); present (1). Patterson, 1982b c12. Jaws, general 289. Jaws: absent (0); present (1). Dupret et al., 2014 c254. 290. Cosmine-like tissue in oral cavity: absent (0); present (1). Friedman, 2007a c56. Dermal lower jaw bones 291. Dermal jaw plates on biting surface of jaw cartilages: absent (0); present (1). Brazeau, 2009 c48. 292. Dentary: absent (0); present (1). New. 293. Large ventromesially directed flange of symphysial region of mandible: absent (0); present (1). Friedman, 2007b c156. 294. Flange like extension of mandible composed of prearticular and Meckelian ossification: absent (0); present (1). Friedman, 2007b c159. 295. Strong ascending flexion of symphysial region of mandible: absent (0); present (1). Friedman, 2007b c155. 296. Labial pit: absent (0); present (1). Cloutier and Ahlberg, 1996 c80. 297. Infradentary: absent (0); present (1). Zhu et al., 2013 c204. 298. Number of infradentaries: one (0); two (1); more than 2 (2). Friedman, 2007b c54; King et al., 2017 c381. 299. Extent of infradentaries: along much of ventral margin of dentary (0); restricted to posterior half of dentary (1). Giles et al., 2015c c93. 300. Infradentary foramina: present (0); absent (1). Ahlberg and Johanson, 1998b 15; King et al., 2017 350. 301. Anterior end of prearticular: far from jaw symphysis (0); near jaw symphysis (1). Zhu and Schultze, 2001 c93. 302. Prearticular - dentary contact: present (0); absent (1). Cloutier and Ahlberg, 1996, c96. 303. Principal coronoid: absent (0); present (1). Cloutier, 1996 c95. 304. Coronoids: present (0); absent (1). Schultze and Cumbaa, 2001 c46. 305. Number of coronoids: more than three (0); three (1). Ahlberg and Clack, 1998a c4, Zhu et al., 2009 c93. 306. Meckelian bone exposed immediately anterior to first coronoid: yes (0); no (1). Ahlberg and Clack, 1998a c22. 307. Submandibulars: absent (0); present (1). Cloutier and Ahlberg, 1996 c104. Palatoquadrate 308. Large otic process of the palatoquadrate: absent (0); present (1). Brazeau, 2009 c49. 309. Insertion area for jaw adductor muscles on palatoquadrate: ventral (0); lateral (1). Brazeau, 2009 c50. 310. Oblique ridge or groove along medial face of palatoquadrate: absent (0); present (1). Brazeau, 2009 c52. 311. Perforate or fenestrate anterodorsal (metapterygoid) portion of palatoquadrate: absent (0); present (1). Brazeau, 2009 c54. 312. Processus ascendens of palatoquadrate: absent (0); present (1). King et al., 2017 c389. 313. Fenestration of palatoquadrate at basipterygoid articulation: absent (0); present (1). Brazeau, 2009 c53. 314. Metapterygoid with developed medial ventral protrusion: absent (0); present (1). Zhu et al., 2013 c216. 315. Palatoquadrate fused with neurocranium: absent (0); present (1). Giles et al., 2015a c101. 316. Jugular vein passes through cranioquadrate passage: absent (0); present (1). King et al., 2017 c126. 317. Autopalatine and quadrate: comineralized (0); separate mineralizations (1). Miles and Dennis, 1979 c22, Giles et al., 2015b c97. 318. Hyosuspensory eminence on quadrate: absent (0); present (1). Friedman, 2007a, c55. 319. Dermal plates on mesial (lingual) surfaces of Meckels cartilage and palatoquadrate: absent (0); present (1). Zhu et al., 2013 c213. 320. Contact between palatoquadrate and dermal cheek bones: continuous contact of metapterygoid and autopalatine (0); metapterygoid and autopalatine contacts separated by gap between commissural lamina of palatoquadrate and cheek bones (1). Zhu et al., 2013 c215. 321. Number of fang pairs on ectopterygoid: one (0); two (1); none (2). Lu et al., 2012 c103. 322. Proportions of entopterygoid: anterior end level with processus ascendens (0); anterior end considerably anterior to processus ascendens (1). Lu et al., 2012 c104. Meckel’s cartilage etc 323. Bilateral series of labial cartilages: absent (0); present (1). King et al., 2017 c393. 324. Pronounced dorsal process on Meckelian bone or cartilage: absent (0); present (1). Hanke and Wilson, 2004 c11; Brazeau, 2009 c55. 325. Adductor fossa: open (0); reduced to narrow slot (1). Schultze, 2001 c69; Ahlberg et al., 2006 c41. 326. Preglenoid process: absent (0); present (1). Davis et al., 2012 c52. 327. Biconcave glenoid on lower jaw: absent (0); present (1). Friedman and Brazeau, 2010 c17; Zhu et al., 2013 c214. 328. Jaw articulation located on rearmost extremity of mandible: absent (0); present (1). Davis et al., 2012 c53. 329. Retroarticular process: absent (0); present (1). Lu et al., 2012 c163. 330. Symplectic articulation: absent (0); present (1). Friedman, 2007b c160. Scapulocoracoid 331. Scapular process of shoulder endoskeleton: absent (0); present (1). Brazeau, 2009 c105. 332. Ventral margin of separate scapular ossification: horizontal (0); deeply angled (1). Brazeau, 2009 c107. 333. Cross sectional shape of scapular process: flattened or strongly ovate (0); subcircular (1). Brazeau, 2009 c108. 334. Flange on trailing edge of scapulocoracoid: absent (0); present (1). Brazeau, 2009 c109. 335. Scapular process with posterodorsal angle: absent (0); present (1). Davis et al., 2012 c114. 336. Endoskeletal postbranchial lamina on scapular process: present (0); absent (1). Brazeau, 2009 c110. 337. Mineralization of internal surface of scapular blade: mineralised all around (0); unmineralised on internal face forming a hemicylindrical cross-section (1); unmineralised on lateral face forming a hemicylindrical cross-section (2). Brazeau, 2009 c111. According to the Burrow and Rudkin, 2014 description, Nerepisacanthus has a scapulocroacoid mineralised only on the medial face. This necessitates a new character state (2). 338. Coracoid process: absent (0); present (1). Brazeau, 2009 c112. 339. Procoracoid mineralization: absent (0); present (1). Brazeau, 2009 c114. 340. Fin base articulation on scapulocoracoid: stenobasal (0); eurybasal (1). Brazeau, 2009 c113. 341. Perforate propterygium: absent (0); present (1). Patterson, 1982b c15. 342. Endoskeletal supports in pectoral fin: multiple elements articulating with girdle (0); single element (‘humerus’) articulating with girdle (1). Zhu and Schultze, 2001 c175. 343. Triradiate scapulocoracoid: absent (0); present (1). Zhu and Schultze, 2001 c171. 344. Subscapular foramen: absent (0); present (1). Zhu and Schultze, 2001 c173. 345. Pectoral propterygium: absent (0); present (1). Zhu and Schultze, 2001 c176. 346. Horizontal plate of scapulocoracoid: absent (0); present (1). Patterson, 1982b c17; Friedman and Blom, 2006 c40. Dermal shoulder girdle 347. Macromeric dermal shoulder girdle: present (0); absent (1). Brazeau, 2009 c99. 348. Dermal shoulder girdle forming a complete ring around the trunk: present (0); absent (1). Brazeau, 2009 c101. 349. Median dorsal plate: absent (0); present (1). Brazeau, 2009 c103. 350. Number of MD plates: one (0); two (1); three (2). Trinajstic and Long, 2009 c445; King et al., 2017 c445. 351. Pronounced internal crista (keel) on median dorsal surface of shoulder girdle: absent (0); present (1). Brazeau, 2009 c104. 352. Posteriorly spine on MD plate: absent (0); present (1). Carr and Hlavin, 2010 c37. 353. Anocleithrum: element developed as postcleithrum (0); element developed as anocleithrum sensu stricto (1). Gardiner and Schaeffer, 1989 cB2. 354. Anocleithrum sensu stricto: exposed (0); subdermal (1). Cloutier and Ahlberg, 1996 c112. 355. Pectoral fenestra completely encircled by dermal shoulder armour: present (0); absent (1). Brazeau, 2009 c102. 356. Dermal shoulder girdle composition: ventral and dorsal (scapular) components (0); ventral components only (1). Brazeau, 2009 c100. 357. Dorsal cleithrum (AL of the Placodermi), ventral cleithrum (AVL of the Placodermi) and pectoral spine (SP of the Placodermi): not fused (0); fused (1). Cloutier and Ahlberg, 1996 c161. 358. Relationship of clavicle to cleithrum: ascending process of clavicle overlapping cleithrum laterally (0); ascending process of clavicle wrapping round anterior edge of cleithrum, overlapping it both laterally and mesially (1). Cloutier and Ahlberg, 1996 c116. 359. Shape of dorsal blade of dermal shoulder girdle (either cleithrum or anterolateral plate): spatulate (0); pointed (1). Cloutier and Ahlberg, 1996 c115. 360. Chang"s apparatus: absent (0); present (1). King et al., 2017 c444. 361. Clavicles/interolateral plates: large plates, comparable in size to cleithrum (0); reduced to small semilunar plates, paired (1); unpaired semilunar plates (2). Jia et al., 2010 c44; King et al., 2017 c443. 362. Median ventral trunk plate(s): absent (0); present (1). King et al., 2017 c447. 363. Extracleithrum: absent (0); present (1). Forey, 1998 c88. 364. Posterior dorsolateral (PDL) plate or equivalent: absent (0); present (1). Giles et al., 2015a c187. 365. PL and PDL overlap: simple (0); insertion (1). Carr and Hlavin, 2010 c42. 366. Left and right PDL contact below MD: absent (0); present (1). King et al., 2017 c438. 367. PDL plate visible externally: yes (0); no (1). King et al., 2017 c439. Dermal pectoral fin 368. Scapular infundibulum: absent (0); present (1). Giles et al., 2015b c190. 369. Pectoral fin base has large, hemispherical dermal component: absent (0); present (1). Brazeau, 2009 c121. 370. Pectoral fins covered in macromeric dermal armour: absent (0); present (1). Brazeau, 2009 c120. 371. Joint in macromeric armoured pectoral fin: unjointed (0); jointed (1). Jia et al., 2010 c27; King et al., 2017 c441. 372 . Cd1 and Cd2 plates: in contact (0); separated (1). Jia et al., 2010 c28. Pectoral fin endoskeleton 373. Number of basals in polybasal pectoral fins: three or more (0); two (1). Giles et al., 2015b c202. 374. Number of mesomeres in metapterygial axis: five or fewer (0); seven or more (1). Cloutier and Ahlberg, 1996 123. 375. Biserial pectoral fin endoskeleton: absent (0); present (1). Giles et al., 2015b c205. 376. Filamentous extension of pectoral fin from axillary region: absent (0); present (1). Giles et al., 2015b c207. 377. Entepicondyle on humerus: present (0); absent (1). King et al., 2017 c418. 378. Distal articulation of propterygium: with fin rays (0); with a second enlarged plate (1); no articulation (2). King et al., 2017 c420. Pelvic fins and claspers 379. Pelvic fins: absent (0); present (1). Brazeau, 2009 c117. 380. Pelvic girdle with substantial dermal component: yes (0); no (1). Zhu et al., 2013 c252. 381. Claspers with large dermal J-shaped element: absent (0); present (1). Long et al., 2015 c258. 382. Claspers: absent (0); present (1). Brazeau, 2009 c119. Removed 'pelvic' from the definition, as placoderm claspers may not be associated with the pelvic fins (Trinajstic et al., 2015). However, for the purposes of this phylogenetic analysis, the claspers of placoderms and chondrichthyans can be considered primary homologues, as opposed to the coding in Long et al., 2015 and King et al., 2017. Fin spines 383. Paired fin spines: absent (0); present (1). Brazeau, 2009 c125. 384. Pectoral fin spine small (bivalve-like): no (0); yes (1). King et al., 2017 c449. 385. Pelvic fin spine: absent (0); present (1). Zhu et al., 2013 c253. 386. Dorsal fin spines: absent (0); present (1). Brazeau, 2009 c123. 387. Anal fin spine: absent (0); present (1). Brazeau, 2009 c124. 388. Median fin spine insertion: shallow, not greatly deeper than dermal bones/scales (0); deep (1). Brazeau, 2009 c126. 389. Intermediate fin spines: absent (0); present (1). Brazeau, 2009 c127. 390. Intermediate spines when present: one pair (0); multiple pairs (1). Giles et al., 2015c c219. 391. Intermediate spines with finlets: absent (0); present (1). King et al., 2017 c481. 392. Prepectoral fin spines: absent (0); present (1). Brazeau, 2009 c128. 393. Prepectoral spines form ‘necklace’: no (0); yes (1). King et al., 2017 c483. 394. Median ventral prepectoral spine: absent (0); present (1). King et al., 2017 c482. 395. Fin spine cross-section: Round or horseshoe shaped (0); Flat-sided, with rectangular profile (1). Giles et al., 2015c c218. 396. Fin spines with ridges: absent (0); present (1). Brazeau, 2009 c129. 397. Fin spines with nodes: absent (0); present (1). Brazeau, 2009 c130. 398. Fin spines with rows of large retrorse denticles: absent (0); present (1). Davis et al., 2012 c134. 399. Expanded spine rib on leading edge of spine: absent (0); present (1). Giles et al., 2015c c224. 400. Spine ridges: converging at the distal apex of the spine (0); converging on leading edge of spine (1). Giles et al., 2015c c225. Median fins 401. Number of dorsal fins, if present: one (0); two (1). Coates and Sequeira, 2001 c10. 402. Posterior dorsal fin shape: base approximately as broad as tall, not broader than all of other median fins (0); base much longer than the height of the fin, substantially longer than any of the other dorsal fins (1). Giles et al., 2015a c229. 403. Basal plate in dorsal fin: absent (0); present (1). Giles et al., 2015b c230. 404. Branching radial structure articulating with dorsal fin basal plate: absent (0); present (1). Giles et al., 2015c c231. 405. Anal fin: absent (0); present (1). Brazeau, 2009 c134. 406. Basal plate in anal fin: absent (0); present (1). Giles et al., 2015c c233. 407. Spine-brush complex: absent (0); present (1). King et al., 2017 c479. Caudal fin 408. Horizontal caudal lobe: absent (0); present (1). Sansom, 2009 c70. 409. Triphycercal tail: absent (0); present (1). King et al., 2017 c452. 410. Caudal radials: extend beyond level of body wall and deep into hypochordal lobe (0); restricted to axial lobe (1). Davis et al., 2012 c138. 411. Supraneurals in axial lobe of caudal fin: absent (0); present (1). Giles et al., 2015a c235. 412. Fringing fulcra: absent (0); present (1). Friedman, 2007b c188. 413. Epichordal lepidotrichia in caudal fin: absent (0); present (1). Cloutier and Ahlberg, 1996 c134. Axial skeleton 414. Synarcual: absent (0); present (1). Brazeau, 2009 c132; Davis et al., 2012 c135. 415. Series of thoracic supraneurals: absent (0); present (1). Cloutier and Ahlberg, 1996 c137. Fin webs 416. Longitudinal scale alignment in fin webs: present (0); absent (1). Giles et al., 2015a c13. 417. Differentiated lepidotrichia: absent (0); present (1). Giles et al., 2013 c14. 418. Interlocking lepidotrichial segments: absent (0); present (1). Friedman, 2007b c187. Postcranial plates 419. Scute-like ridge scales (basal fulcra): absent (0); present (1). Patterson, 1982b c19. 420. Longitudinal rows of enlarged keeled scutes: absent (0); present (1). King et al., 2017 c484. 421. Series of median hexagonal scutes anterior to first dorsal fin: absent (0); present (1). King et al., 2017 c480. Scales 422. Body scale growth pattern: monodontode (0); polyodontode (1). Brazeau, 2009 c8. 423. Body scale growth concentric: absent (0); present (1). Brazeau, 2009 c9. 424. Postcranial scales with areal or appositional growth crowns: absent (0); present (1). Burrow et al., 2016 c260. 425. Body scales with peg-and-socket articulation: absent (0); present (1). Coates, 1999 c3. 426. Peg on rhomboid scale: narrow (0); broad (1). Patterson, 1982b c5. 427. Anterodorsal process on scale: absent (0); present (1). Patterson, 1982b c4. 428. Body scale profile: distinct crown and base demarcated by a constriction (neck) (0); flattened (1). Brazeau, 2009 c11. 429. Profile of scales with constriction between crown and base: neck similar in width to crown (0); neck greatly constricted, resulting in anvil-like shape (1). Giles et al., 2015c c22. 430. Body scales with bulging base: absent (0); present (1). Brazeau, 2009 c12. 431. Body scales with flattened base: present (0); absent (1). Brazeau, 2009 c13. 432. Scales: macromeric (0); micromeric (1). Friedman and Blom, 2006 c34. 433. Flank scales alignment: vertical rows (0); oblique rows or hexagonal/rhombic packing (1); disorganised (2). Davis et al., 2012 c14. 434. Basal pore in scales: absent (0); present (1). Giles et al., 2015c c25. 435. Scales with well developed pores on ganoine surface: absent (0); present (1). Friedman and Blom, 2006 c35. Sensory lines, general 436. Sensory line network: preserved as open grooves (0); pass through canals enclosed within dermal bones (1). Brazeau, 2009 c40. 437. Sensory canals/grooves: contained within the thickness of dermal bones (0); contained in prominent ridges on visceral surface of bone (1). Giles et al., 2015c c31. Sensory lines, snout 438. Course of ethmoid commissure: middle portion through median rostral (0); sutural course (1); through bone center of premaxillary (2). Zhu and Schultze, 2001 c43. 439. Ethmoid commissure fused into midline canal: absent (0); present (1). King et al., 2017 c320. 440. Infraorbital canal follows premaxillary suture: no (0); yes (1). Cloutier and Ahlberg, 1996 c100. 441. Anterior supraorbital canal: absent (0); present (1). Zhu and Gai, 2007 c38; King et al., 2017 c309. 442. Semicircular pit-line: absent (0); present (1). Jia et al., 2010 c23. 443. Supraorbital sensory canal: absent (0); present (1). Zhu and Gai, 2007 c39; King et al., 2017 c307. 444. Course of supraorbital canal: between anterior and posterior nostrils (0); anterior to both nostrils (1). Cloutier and Ahlberg, 1996 c98. 445. Contact of supraorbital and infraorbital canals: in contact rostrally (0); not in contact rostrally (1). Zhu et al., 2001 c34. 446. Contact between otic and supraorbital canals: not in contact (0); in contact (1). Cloutier and Ahlberg, 1996 c102. 447. Branching sensory canal system at posterior of supraorbital canals: absent (0); present (1). King et al., 2017 c306. 448. Posterior end of supraorbital canal: close to posterior and middle pit-lines (0); anterior to posterior and middle pit-lines (1); extends posterior to middle and posterior pit-lines (2). King et al., 2017 c292. 449. Anterior pit-line of dermal skull roof: absent (0); present (1). Giles et al., 2015a c34. 450. Position of anterior pit-line: on paired median skull roofing bones over the otico-occipital division of braincase (0); on paired median skull roofing bones over the sphenoid division of braincase (1). Cloutier and Ahlberg, 1996 c103. 451. Course of supraorbital canal: straight (0); lyre-shaped (1). Zhu et al., 2013 c184. 452. Median dorsal canal: absent (0); present (1). Zhu and Gai, 2007 c43; King et al., 2017 c310. Sensory lines, skull roof posterior 453. Infra-orbital sensory line: crosses lateral field (0); does not cross lateral field (1). Sansom, 2009 c31. 454. Festooned pattern of sensory canals: absent (0); present (1). Zhu and Gai, 2007 c2. 455. Branching of lateral transverse canal: absent (0); present (1). Zhu and Gai, 2007 c39. 456. Supraorbital canals and posterior pitlines cross as an X: no (0); yes (1). Long et al., 2015 c256; King et al., 2017 c300. 457. Otic canal: runs through skull roof (0); follows edge of skull roof (1). Ahlberg and Johanson, 1998b c66. 458. Extension of otic canal beyond infraorbital canal (‘P’ canal): absent (0); present (1). King et al., 2017 c319. 459. Otic canal extends through postparietals: absent (0); present (1). Cloutier and Ahlberg, 1996 c101. 460. Otic canal runs along mesial margin of marginal plate: absent (0); present (1). Dupret et al., 2009 c16. 461. Central sensory lines: absent (0); present (1). Dupret et al., 2009 c31. 462. Junction of posterior pitline and main lateral line: far in front of posterior margin of skull roof (0); close to posterior margin of skull roof (1). Zhu et al., 2013 c166. 463. Middle and posterior pit-lines on postparietal: posteriorly situated (0); mesially situated (1). Zhu et al., 2001 c39. 464. Position of middle and posterior pit-lines: close to midline (0); near the central portion of each postparietal (1). Zhu et al., 2006 c41. 465. Posterior pitline and postmarginal canal: staggered (0); confluent (1). King et al., 2017 c321. 466. Postmarginal canal: absent (0); present (1). King et al., 2017 c315. 467. Sensory line commissure across extrascapular bones: absent (0); present (1). King et al., 2017 c323. Sensory lines, cheek 468. Preopercular canal: absent (0); present (1). King et al., 2017 c316. 469. Preopercular canal meets main canal: no (0); yes (1). King et al., 2017 c317. 470. Horizontal sensory canal: absent (0); present (1). King et al., 2017 c314. 471. Jugal portion of infraorbital canal joins supramaxillary canal: present (0); absent (1). Davis et al., 2012 c17. 472. Supraoral canal: absent (0); present (1). King et al., 2017 c318. 473. Sensory canal or pit-line associated with maxilla: absent (0); present (1). Friedman, 2007b c152. Sensory lines, jaw 474. Course of mandibular canal: not passing through most posterior infradentary (0); passing through most posterior infradentary (1). Cloutier and Ahlberg, 1996 c111. 475. Course of mandibular canal: passing through dentary (0); not passing through dentary (1). Patterson, 1982a c7. Sensory lines, postcranial 476. Dorsal branch of main lateral line canal on PDL: absent (0); present (1). King et al., 2017 c325. 477. Sharp downward bend on PDL plate sensory line: absent (0); present (1). King et al., 2017 c326. 478. Sensory line canal: passes between or beneath scales (0); passes over scales and/or is partially enclosed or surrounded by scales (1); perforates and passes through scales (2). Friedman and Brazeau, 2010 c26; Davis et al., 2012 c15. Visceral skeleton 479. Foramen in hyomandibular: absent (0); present (1). Friedman, 2007b c163. 480. Endoskeletal hyoid rays: absent (0); present (1). King et al., 2017 c150. 481. Anteriormost unpaired element of branchial skeleton contacted by: one branchial arch only (0); two or more branchial arches (1). Dearden et al., 2019 c73. 482. Multiple unpaired branchial mineralizations: absent (0); present (1). Dearden et al., 2019 c74. 483. Interhyal: absent (0); present (1). Davis et al., 2012 c38. 484. Hypohyal: absent (0); present (1). Giles et al., 2015c c75. 485. Endoskeletal urohyal: absent (0); present (1). Friedman, 2007b c164. 486. Gill skeleton extends posteriorly beyond occiput: absent (0); present (1). Dearden et al., 2019 c67. 487. Sublingual rod: absent (0); present (1). King et al., 2017 c149. 488. Disposition of the interbranchial ridges of the oralobranchial chamber roof: oligobranchiate (0); orthobranchiate (1); nectaspidiform (2). Sansom, 2009 c62. 489. Number of branchial fossae: 5–7 (0); 9–17 (1); more than 20 (2). Zhu and Gai, 2007 c49. Homology-variable characters The following characters have variable homology and the data matrix presents two options for their codings in placoderms. Both the marginal jaw bones (characters 490–498) and the palatal bones (characters 599–507) have the same set of dependent characters. Characters regarding the external, facial part of the maxilla and premaxilla are dependent on the presence of a facial lamina, which is absent in placoderms. These characters therefore are coded as inapplicable in placoderms regardless of homology, and do not need to be included in the homology-variable characters. 490. Premaxilla: absent (0); present (1). New. 491. Maxilla: absent (0); present (1). Cloutier and Ahlberg, 1996 c19. 492. Facial laminae on (pre)maxilla: absent (0); present (1). New. 493. Palatal laminae on (pre)maxilla: absent (0); present (1). New. 494. Premaxilla fused in midline: absent (0); present (1). King et al., 2017 c372. 495. Pipe-like ridges on (pre)maxilla: absent (0); present (1). King et al., 2017 c373. 496. Premaxilla posterior process: absent (0); present (1). New. 497. Fangs on premaxilla palatal lamina: absent (0); present (1). New. 498. Maxilla fragmented into multiple bones: absent (0); present (1). New. 499. Vomers: absent (0); present (1). New. 500. Dermopalatine: absent (0); present (1). New. 501. Facial laminae on vomer/dermopalatines: absent (0); present (1). New. 502. Palatal laminae on vomer/dermopalatines: absent (0); present (1). New 503. Vomers fused in midline: absent (0); present (1). King et al., 2017 c372. 504. Pipe-like ridges on vomer/dermopalatines: absent (0); present (1). King et al., 2017 c373. 505. Vomer posterior process: absent (0); present (1). Lu et al., 2012 c89; Carr and Hlavin, 2010 c68; King et al., 2017 c375. 506. Fangs on vomer palatal lamina: absent (0); present (1). Adapted from Ahlberg and Johanson, 1998b c24. 507. Dermopalatine fragmented into multiple bones: absent (0); present (1). Lu et al., 2012 c106; King et al., 2017 c378.
